# Testing cellular provenance in the cancer extracellular matrix

**DOI:** 10.3389/fcell.2026.1984128

**Published:** 2026-10-01

**Authors:** Mingtao Ma, Yihan Huang, Enni Tang, Yueying Zhang, Xixi Yang, Lin Huang, Hu Ma

**Affiliations:** 1 Department of Oncology, The Second Affiliated Hospital of Zunyi Medical University, Zunyi, China; 2 Department of Histology and Embryology, Zunyi Medical University, Zunyi, China

**Keywords:** cellular provenance, collagen I, extracellular matrix, fibronectin, tenascin C, tumor microenvironment

## Abstract

Composition, architecture, and mechanics are central descriptors of the tumor extracellular matrix (ECM), but they do not identify the cells that synthesized and deposited it. Cellular provenance should therefore be distinguished from production context, deposition context, post-secretory history, and final matrix state. The central causal question is whether producer identity or state creates a persistent functional difference after abundance, proteoform, location, processing, assembly, and receptor environment are accounted for. Current evidence remains compatible with three explanations. The producer-mediated determinant model invokes source-linked proteoforms, co-secretomes, deposition patterns, or assembly routes. The provenance-associated model treats producer identity as a marker of abundance, timing, location, or cell state. The provenance-independent model predicts functional equivalence once molecular and matrix states are matched. Studies of collagen I, tenascin C, and fibronectin reveal three recurring patterns: producer-linked proteoform differences, stage-dependent source replacement, and source redundancy or assembly dependence. Protein-level source attribution and source-specific necessity are increasingly feasible, but abundance- and location-matched reciprocal source exchange remains uncommon. Cellular provenance should therefore be treated as a falsifiable variable whose functional significance is traced to a measurable molecular, spatial, mechanical, or assembly mediator.

## 1 Introduction

The extracellular matrix (ECM) is a dynamic component of tumor biology rather than an inert scaffold. Its proteins and regulators provide structural continuity, present ligands, store growth factors, transmit force, and guide vascular, immune, and tumor-cell movement. Matrisome definitions and ECM-enrichment proteomics made this complexity experimentally tractable (Naba et al., 2012). Imaging and mechanical studies subsequently linked matrix organization and mechanics to tumor initiation, invasion, metastasis, and progression (Provenzano et al., 2008; Levental et al., 2009; Acerbi et al., 2015; Fan et al., 2024). These advances moved the field beyond a descriptive view of desmoplasia.

Bulk assays report composition and abundance, whereas spatial measurements add location. Neither identifies the cells that synthesized and deposited protein that persists at a site. More generally, mRNA abundance is an imperfect predictor of protein abundance because mRNA turnover, translational efficiency, and protein degradation introduce additional regulatory layers (Vogel and Marcotte, 2012; Liu et al., 2016). For ECM proteins, secretion, extracellular processing, insolubilization, and retention add further separation between transcript abundance and mature matrix deposition. A cell assigned a fibronectin 1 (FN1) or collagen type I alpha 1 chain (COL1A1) transcript by single-cell RNA sequencing is therefore a candidate producer, not necessarily the source of mature deposited protein. Protein detection establishes composition, not origin.

The disconnect between expression and matrix history is biologically consequential. A shared gene symbol can conceal alternative transcripts, modified proteoforms, oligomers, or networks. Conversely, identical products can occupy different neighborhoods, encounter different proteases and forces, or engage different receptors. Total abundance therefore cannot capture architecture, mechanics, ligand accessibility, or local responder programs. Proteomic and temporal matrisome studies show that ECM protein abundance can diverge from corresponding transcriptional states and change dynamically during tumor progression (Naba et al., 2014; Papanicolaou et al., 2022; Pereira et al., 2024).

Single-cell and spatial transcriptomics identify candidate producer states. ECM-enrichment proteomics and species-resolved mass spectrometry provide protein- or compartment-level evidence, whereas imaging resolves deposits and mechanics. Because each method leaves part of the chain unresolved, credible source claims require complementary measurements. This is especially important for broad lineages, blood-borne proteins, and matrix that outlives its producers.

Current evidence permits three interpretations. Producer identity may create a persistent molecular, co-secretory, spatial, or assembly difference. Alternatively, identity may only track abundance, timing, location, cell state, or later remodeling. A third possibility is that products become interchangeable once molecular, spatial, mechanical, and receptor states are matched. Collagen I, tenascin C, and fibronectin are informative comparisons because each has multiple sources, context-dependent phenotypes, and unresolved cross-source equivalence. Examples from the literature map onto these three models: PDAC collagen I provides a producer-linked proteoform example, tenascin C illustrates stage-dependent source replacement, and fibronectin illustrates source redundancy and assembly dependence (Chen et al., 2022; Oskarsson et al., 2011; O'Connell et al., 2011; Lugano et al., 2018; Malik et al., 2010).

Testing cellular provenance therefore requires a causal rather than annotational standard. The question is whether producer identity or state creates a persistent functional difference after molecular and matrix context are accounted for. This distinction matters for metastasis, biomarkers, and therapeutic compensation, while leaving provenance-associated and provenance-independent outcomes possible. Figure 1 summarizes the conceptual framework and competing causal models.

**FIGURE 1:**
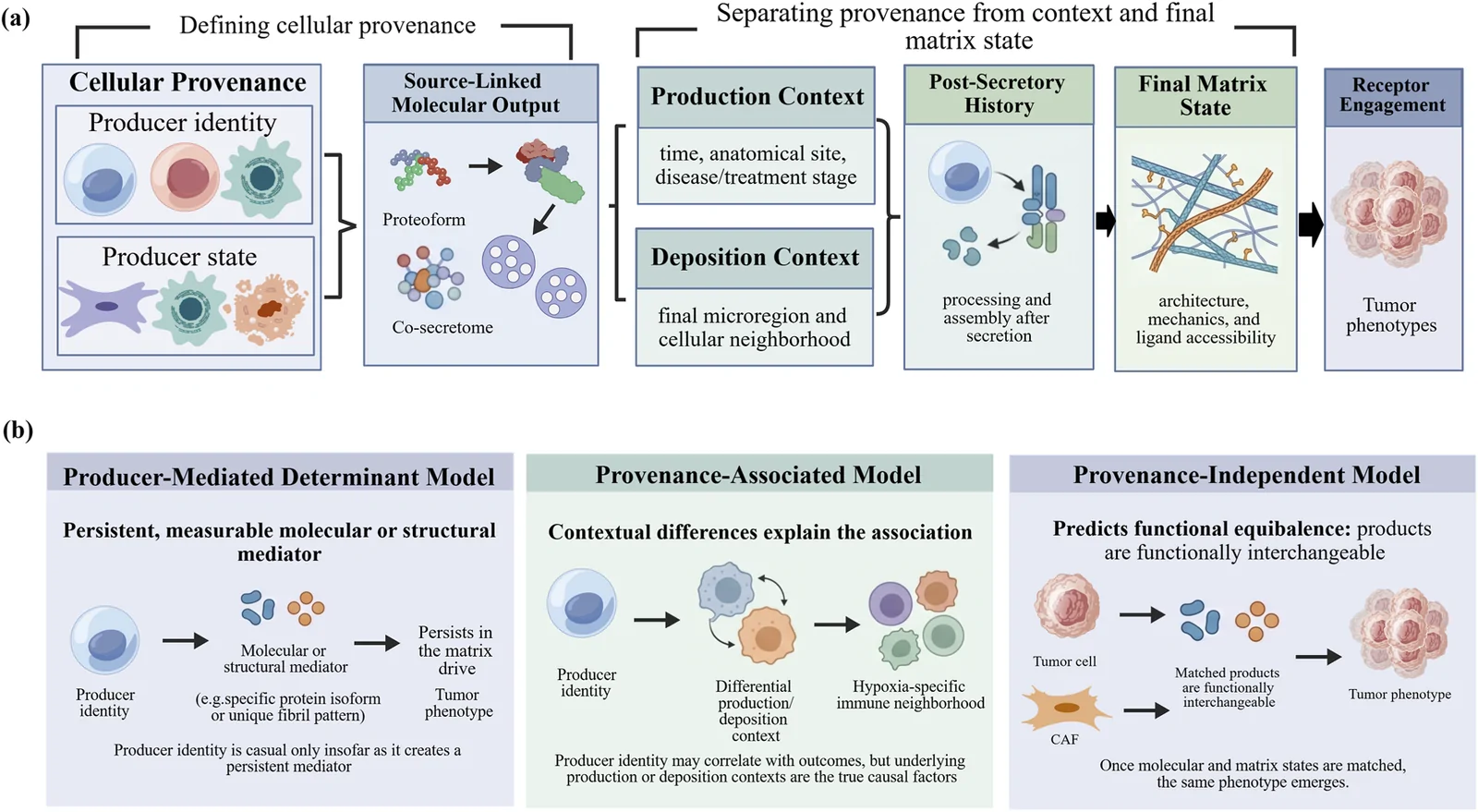
Producer identity, matrix history, and competing causal models of ECM function. **(a)** Conceptual framework separating cellular provenance, defined by producer identity and state, from source-linked molecular output, production and deposition contexts, post-secretory history, final matrix state, and receptor engagement. These successive layers connect matrix production to tumor phenotypes. **(b)** Three competing causal models of extracellular matrix (ECM) function: the producer-mediated determinant model, in which producer identity generates a persistent molecular or structural mediator; the provenance-associated model, in which differences in production or deposition context account for the observed phenotype; and the provenance-independent model, in which products are functionally interchangeable once relevant molecular and matrix states are matched. CAF, cancer-associated fibroblast. Created with BioGDP.com (Jiang et al., 2025).

## 2 Producer identity, matrix history, and matrix state

### 2.1 From expression to remodeling

A gene-expressing cell contains a detectable transcript. A protein-producing cell translates the relevant polypeptide, a protein-secreting cell exports it, and a matrix-depositing cell contributes it to an insoluble or functionally organized extracellular compartment. These categories often overlap, but none guarantees the next. Secreted procollagen can be degraded before fibril incorporation, soluble fibronectin can circulate without local fibrillogenesis, and a basement-membrane component can move before deposition. A provenance statement should therefore specify whether the evidence concerns RNA, intracellular or soluble protein, nascent secretion, or deposited ECM.

Matrix assembly is a distinct role. Fibronectin fibrillogenesis requires integrin-mediated traction and partner interactions, so the cell that organizes a fiber need not be its sole fibronectin producer. In glioma, CD93-MMRN2 signaling controlled β1-integrin activation and vascular fibronectin fibrillogenesis without changing FN1 transcription (Lugano et al., 2018), separating matrix assembly from gene expression. Cancer-associated fibroblasts (CAFs) can align fibronectin into migration-supporting tracks through contractility and receptor signaling (Attieh et al., 2017; Erdogan et al., 2017), while carcinoma cells can migrate collectively along fibronectin-rich interfaces (Gopal et al., 2017). Calling all of these cells “the source” would obscure the distinction between supplying material and organizing it.

Remodeling is likewise distinct. LOX-family enzymes can crosslink collagen made elsewhere, proteases can expose cryptic sites or release matrikines, and cells can orient pre-existing fibers or modify proteoglycans. In pancreatic ductal adenocarcinoma (PDAC), collagenolysis-dependent discoidin domain receptor 1 (DDR1) signaling shows that receptor output can depend strongly on the processed state of collagen (Su et al., 2022). Collagen abundance alone may therefore be insufficient to predict function. A rigorous report should name the producer, depositor, assembler, and remodeler only to the extent that each role is directly supported.

### 2.2 Provenance and matrix context

“Fibroblast-derived collagen” is often too coarse to be reproducible, but greater precision requires distinct terms rather than a broader definition of provenance. Cellular provenance specifies producer identity and state. Production context specifies when and where synthesis occurred, including the disease or treatment stage. Deposition context specifies the final microregion, neighboring cells, available receptors, and local protease and mechanical environment. Post-secretory history includes proteolysis, crosslinking, traction, alignment, assembly, degradation, and incorporation into networks. Final matrix state describes the proteoform, architecture, fiber orientation, porosity, stiffness, viscoelasticity, ligand accessibility, age, and receptor-readable configuration encountered by responder cells.

These categories can covary but are not synonymous. A myofibroblastic CAF and an inflammatory fibroblast can occupy distinct production contexts despite sharing a broad fibroblast identity (Öhlund et al., 2017). Endothelial fibronectin produced during angiogenic sprouting can enter a perivascular network (Lugano et al., 2018; Murphy et al., 2015), whereas hepatocyte-derived plasma fibronectin reaches tumors through the circulation and vascular leakage (Malik et al., 2010; von Au et al., 2013). Single-cell data may justify the statement “transcriptionally attributed to a myofibroblast state.” Species-specific peptides support “human tumor-cell versus mouse host contribution,” whereas a conditional allele supports a lineage requirement if deposition is measured. Claims should not exceed the resolution of the assay.

### 2.3 Gene, proteoform, and network identity

The phrase “the same ECM molecule” conceals several levels of identity. Products can share a gene identity yet differ in alternative splicing, promoter use, signal peptide, glycosylation, hydroxylation, proteolytic cleavage, disulfide pattern, or crosslinking. Fibronectin containing extra domain A or B is not molecularly identical to a plasma isoform. Collagen I α1 homotrimers are not the same proteoform as α1/α2 heterotrimers. A cleaved collagen fragment that engages a receptor is not equivalent to intact fibrillar collagen. At the next level, an identical purified polypeptide in solution, adsorbed to plastic, assembled into a fiber, or incorporated into a crosslinked network represents a different supramolecular state.

The relevant experimental comparison must therefore be specified. A study may show functional non-equivalence between products with the same gene identity but different proteoforms. This supports a source-linked mechanism when producer biology generates the proteoform difference, but not an effect independent of proteoform. In PDAC, malignant cells predominantly produced collagen I α1 homotrimers, whereas fibroblasts produced conventional α1/α2 heterotrimers. The purified forms differed in integrin-dependent signaling (Chen et al., 2022). These results support a producer-mediated determinant mechanism through proteoform identity, not the idea that molecularly matched collagen differs merely because another cell produced it. If differences persist after measured states are matched, an unmeasured molecular or structural mediator should be sought instead of invoking an abstract “source memory.”

### 2.4 Multicellular matrix assembly

An individual protein can sometimes be assigned to one producer, but an ECM fiber or microdomain is usually a collective product. One cell may supply fibronectin, another collagen, a third LOX, a fourth a protease inhibitor, and a fifth the integrin traction that organizes the network. CAF-derived extracellular-vesicle LOX (Liu et al., 2023), fibroblast-derived LOX in cholangiocarcinoma (Lewinska et al., 2024), and macrophage-derived OSM induction of lysyl oxidase-like 2 (LOXL2) in PDAC cells (Alonso-Nocelo et al., 2023) illustrate how multiple cell types can directly or indirectly regulate collagen remodeling. Network-level provenance should therefore allow source convergence, with multiple producers contributing the same or different components to a common matrix microregion.

Basement membranes and vascular niches are especially complex because endothelial cells, pericytes, fibroblasts, and tumor cells may supply different chains while remodeling the same barrier. Describing a vascular basement membrane as “endothelial-derived” is therefore rarely adequate. The source of a chain, the cell that assembles the network, and the cell that breaches it during intravasation may all differ.

### 2.5 Local, systemic, and historical sources

Not all tumor-associated matrix originates locally. Hepatocyte-derived plasma fibronectin, liver-derived fibrinogen, platelet cargo, and circulating matrix regulators can enter tumors or metastatic sites. Genetic loss of plasma fibronectin reduced lung metastasis in a host model, whereas circulating fibronectin has shown context-dependent effects on tumor growth (Malik et al., 2010; von Au et al., 2013). Fibrinogen-deficient mice established causal contributions of a systemic coagulation-associated matrix protein to spontaneous and hematogenous metastasis, including protection of circulating tumor cells from immune elimination (Palumbo et al., 2002; Palumbo et al., 2000; Palumbo et al., 2005). These proteins should not automatically be classified as stable structural tumor ECM, but they show that a local cell map can miss a biologically important systemic source.

Platelets add another layer. Tumor-cell collagen and heat shock protein 47 (HSP47)-dependent platelet interactions, together with platelet-specific growth-factor programs affecting vessel integrity, can alter metastatic transit without identifying a local matrix-producing cell (Xiong et al., 2020; Zhang et al., 2020). In such settings, provenance must distinguish the distant organ that synthesized a circulating protein, the blood cell that stored or released it, and the tumor microregion in which it acted.

Historical sources create a different mismatch. ECM proteins can outlast the producing cell or state. Tissue proteome-turnover measurements show that protein half-lives vary over orders of magnitude, providing a physical basis for matrix legacy (Li et al., 2025). After therapy, involution, or cell-state transition, current populations may not resemble those that deposited the matrix. Aged and irradiated tissue matrices, together with obesity- and diet-induced remodeling, illustrate how earlier tissue states can generate persistent matrix-associated or microenvironmental legacy effects (Yang et al., 2024; Conner et al., 2024; Zhu et al., 2024; Pascual et al., 2021). Source convergence refers to several producers contributing to one microdomain. Source relay separates an initiating signal from a downstream matrix producer, whereas source replacement occurs when another lineage restores a product after the first producer is lost. Matrix legacy denotes persistent material, not a mystical memory of cellular identity.

## 3 Evidence for ECM production and causality

No single method captures every step from transcription to long-lived matrix function. Technologies should therefore be compared along five independent evidence dimensions: (1) source attribution, (2) protein deposition, (3) spatiotemporal localization, (4) source-specific causality, and (5) a mechanism of functional non-equivalence. A method can be strong in one dimension and weak in another. Spatial imaging may define final architecture with high resolution while revealing little about the original producer. Conditional genetics can identify a lineage requirement while leaving proteoform identity and spatial rescue unresolved. Table 1 summarizes representative studies that provide protein-level or causal evidence for tumor ECM provenance.

**TABLE 1:** Representative studies providing protein-level or causal evidence for tumor ECM provenance.

Molecule/proteoform	Producer and context	Source/deposition evidence	Source-specific intervention	Rescue or exchange	Main alternative explanation	Evidence verdict
Collagen I	αSMA-positive myofibroblasts; PDAC progression	Lineage deletion, fibrillar collagen, SHG, stiffness (Chen et al., 2021)	Myofibroblast Col1a1 deletion	No collagen rescue or cross-source exchange	Producer-state effects; incomplete replacement	Strong producer-specific necessity; non-equivalence untested
Collagen I	Malignant cells; PDAC growth/metastasis	Species-aware assays, ECM enrichment, processing evidence (Tian et al., 2021)	Cancer-cell collagen perturbation; BMP1-site mutation	Within-source processing epistasis	Local amount, processing, co-secretome	Strong tumor-cell program; source equivalence unresolved
Collagen I α1 homotrimer versus α1/α2 heterotrimer	PDAC cells versus fibroblasts	Biochemistry plus cell-specific deletion; stromal collagen maintained (Chen et al., 2022)	Cancer-cell Col1a1 deletion	Purified proteoform rescue; no spatially matched *in vivo* swap	Products are different proteoforms	Strong producer-mediated effect through proteoform identity
Tenascin C	Breast cancer cells; early lung colonization	Tumor-cell perturbation and metastatic-site staining (Oskarsson et al., 2011)	Tumor-cell TNC knockdown	No reciprocal stromal exchange	Colonizing-cell state, timing, splice form	Tumor-source necessity in the tested model
Tenascin C	S100A4-positive host stroma; metastatic outgrowth	Stromal-cell manipulation and tissue imaging (O'Connell et al., 2011)	Host population/TNC pathway perturbation	No matched tumor-source exchange	Broad S100A4 lineage, location, immune effects	Host contribution; product equivalence unknown
Tenascin C	Tumor versus host compartments; syngeneic mouse tumors	Source-stratified models with macrophage phenotyping (Deligne et al., 2020)	Tumor/host TNC context; TNC or TLR4 antibodies	No equal-dose, isoform- and location-matched exchange	Co-secretome, splice form, macrophage context	Opposing source-associated immune effects; strict non-equivalence unresolved
Fibronectin	Endothelial/vascular assembly; glioma	Perivascular fibrils; FN1 RNA unchanged after CD93 perturbation (Lugano et al., 2018)	Endothelial CD93/MMRN2 pathway	CD93 re-expression/conditioned-medium rescue; no source swap	Endothelial behavior and assembly, not synthesis alone	Strong assembly evidence; incomplete source attribution
Fibronectin	Endothelium; tumor angiogenesis	Conditional endothelial fibronectin/integrin genetics (Murphy et al., 2015)	Endothelial deletion	No	Plasma/stromal compensation	Important negative evidence for universal source dependence
Plasma fibronectin	Hepatocyte-derived circulating pool; lung metastasis	Host plasma-fibronectin deficiency and lung-associated protein (Malik et al., 2010)	Systemic source loss	No matched local rescue	Coagulation, leakage, inflammation	Causal systemic contribution; local equivalence unknown
Fibronectin	Breast cancer cells; dissemination	Tumor-cell FN1 perturbation and cell-associated matrix (Shinde et al., 2018)	Tumor-cell FN1 loss/restoration	Within-source restoration	Adhesion and malignant-cell state	Context-dependent tumor-source program; no cross-source test
SNED1; LTBP3	Human tumor compartment; metastatic breast xenografts	Species-specific ECM peptides and matrix changes (Naba et al., 2014)	Tumor-cell shRNA	No deposition-matched rescue or exchange	Tumor-cell state, co-secretome, xenograft context	Protein-compartment assignment plus necessity; non-equivalence untested

### 3.1 Single-cell and spatial transcriptomics

Single-cell RNA sequencing resolves candidate producer states, co-expression programs, and disease-associated transitions. Spatial transcriptomics adds neighborhood information and can place an FN1-high fibroblast state near an invasive edge or an endothelial ECM program near a vessel. Representative atlases reveal stromal heterogeneity and spatially restricted matrix programs that bulk expression obscures (Goveia et al., 2020; Wu et al., 2020; Jain et al., 2023; Grünwald et al., 2021; Risom et al., 2022; Heiser et al., 2023; Bansaccal et al., 2023).

However, the direct measurement is RNA. Dropout, ambient RNA, dissociation bias, and uncertain spatial deconvolution complicate even transcriptional attribution. More fundamentally, mRNA turnover, translational efficiency, protein stability, secretion, post-translational modification, extracellular processing, and matrix retention separate transcript abundance from mature ECM deposition (Vogel and Marcotte, 2012; Liu et al., 2016). RNA modifications such as N6-methyladenosine can further regulate transcript stability and translation (Zaccara et al., 2019). Examples from ECM biology make this distinction more concrete. Endothelial CD93 perturbation alters vascular fibronectin fibrillogenesis without changing FN1 transcription (Lugano et al., 2018), while PDAC collagen phenotypes vary with producer-specific processing and proteoform state (Tian et al., 2021; Chen et al., 2022). Spatial RNA location therefore need not match protein deposition. The appropriate conclusion is “a candidate producer state at this time and place.” Claims of mature ECM provenance require protein-level corroboration.

### 3.2 Species-resolved mass spectrometry and ECM-enrichment proteomics

Human–mouse xenografts provide a natural labeling experiment. Species-divergent peptides can assign ECM proteins to human tumor cells or mouse host cells after decellularization or biochemical ECM enrichment. This strategy demonstrated contributions from both compartments and identified tumor-cell-derived sushi, nidogen and EGF-like domains 1 (SNED1) and latent transforming growth factor beta binding protein 3 (LTBP3) in metastatic mammary carcinoma xenografts (Naba et al., 2014). Related analyses have characterized tumor education of the stromal proteome, treatment responses in pancreatic patient-derived xenografts, and species-deconvolved reference libraries (Wang et al., 2017; Wang et al., 2023; Krasny et al., 2020).

The main strength of this method is protein-level attribution, but its resolution is often binary: human tumor versus mouse host. It cannot distinguish a mouse fibroblast from a macrophage, endothelial cell, pericyte, or hepatocyte. Shared peptides may be unassignable or require probabilistic treatment. Sequence homology and database choice affect sensitivity, and tumor-host interactions cross species. Mouse stromal contamination can also distort apparent human tumor proteomes if deconvolution is not explicit (Shi et al., 2023). ECM enrichment introduces additional recovery bias because heavily crosslinked, insoluble, glycosylated, and low-abundance proteins are not extracted equally. The strongest conclusion is therefore often that deposited protein contains species-specific evidence from one compartment, not that a particular cell type produced every copy.

In complex multicellular tissues, source-resolved proteomics should therefore be interpreted as incomplete, abundance-dependent sampling rather than an exhaustive census of the matrix. ECM-enrichment protocols do not achieve complete biochemical separation from intracellular proteins, and different extraction strategies recover distinct subsets of the matrisome (Krasny et al., 2016; McCabe et al., 2021). Residual cytoplasmic or membrane-associated proteins may therefore complicate interpretation of an apparent matrix proteome. ECM extraction is also inherently selective: highly insoluble and crosslinked material can resist conventional extraction, leading to under-representation of structural ECM components (Barrett et al., 2017; McCabe et al., 2021). Abundant or readily ionized peptides are also more likely to be identified than low-abundance species. PTM-rich ECM proteins add another analytical challenge because many matrix proteins are heavily modified, and site-specific glycosylation or other modifications may escape standard bottom-up workflows (Naba, 2023); metabolic-labeling strategies have been developed to improve access to otherwise difficult-to-detect O-linked glycan modifications (Boyce et al., 2011). For multimeric ECM proteins, peptide intensities from individual chains should not be assumed to reconstruct native complex stoichiometry. Accordingly, absence of a source-specific peptide does not establish absence of production, and relative peptide abundance should not be converted directly into absolute producer contribution. In complex multicellular models, proteomic source attribution is most convincing when cross-validated with cell-selective labeling, spatial imaging, or lineage-specific perturbation.

No single analytical platform resolves all of these biases. Combining complementary methods can, however, reduce several of them. Fractionated extraction workflows that analyze cellular and ECM fractions separately can improve matrisome coverage and make cross-compartment carryover more explicit (McCabe et al., 2021). Cell-type-selective metabolic labeling can provide positive evidence of nascent synthesis from defined producers, whereas appropriately designed proximity-labeling approaches can strengthen source assignment; chemoselective enrichment of newly deposited ECM can improve detection of low-abundance nascent matrix proteins (Wei et al., 2021; Kim et al., 2021; Ling et al., 2025). Spatial extracellular proteomics can then map deposition context, and targeted LC–MS/MS with stable-isotope internal standards can provide calibrated quantification of prioritized ECM proteins or proteoforms (Macdonald et al., 2024; Rosin et al., 2024). When paired with source-specific perturbation and rescue, these methods can help identify ECM components whose cellular origin is well supported and whose functional relevance can be tested directly.

Conventional ECM proteomics remains informative without xenograft deconvolution. It identifies deposited matrisome proteins and can track temporal reprogramming or metastasis-associated changes (Papanicolaou et al., 2022; Jailkhani et al., 2023). These data should be paired with cell-resolved hypothesis generation or labeling rather than used to assign each protein retrospectively to the cell type with the highest RNA.

### 3.3 Cell-selective metabolic labeling and protein tags

Cell-type-selective proteomics can extend source attribution beyond the species level. Mutant aminoacyl-tRNA synthetases enable designated cells to incorporate noncanonical amino acids into nascent proteins for bioorthogonal enrichment (Alvarez-Castelao et al., 2017; Wei et al., 2021). Related secretome-labeling approaches can identify lineage-resolved extracellular proteins in tumors and circulation (Kim et al., 2021; Swietlik et al., 2023). These methods measure synthesis or release more directly than RNA and can define temporal windows.

Recent engineering approaches have expanded nascent-matrix analysis beyond bulk secretome measurements. In *ex vivo* lung tissue, local matrix manipulation combined with lineage-traced models enabled spatial analysis of nascent protein deposition and remodeling under controlled mechanical conditions (Ahmed et al., 2025). A glycosylation-enabled chemoselective workflow enriched newly synthesized ECM in bioengineered tumor tissues and revealed remodeling events that were under-represented by bulk proteomics (Ling et al., 2025). During murine forelimb development, three-dimensional mapping resolved the timing and architecture of ECM deposition at the developing muscle-tendon interface (Leng et al., 2025). These approaches improve temporal or spatial resolution of newly deposited matrix and could be combined with cell-selective labeling to strengthen producer assignment. By themselves, however, they do not yet provide complete cell-of-origin attribution in complex tumors.

For ECM, the analytical bottleneck lies downstream. A labeled nascent protein recovered from soluble fluid is not necessarily a deposited matrix protein. Labels or tags may alter secretion, multimerization, cleavage, or receptor binding. Low incorporation can be difficult to detect in slowly turning-over ECM, and enrichment methods optimized for soluble proteomes may miss insoluble, crosslinked material. Proximity labeling identifies residence near the enzyme rather than unambiguously identifying synthesis. Decisive validation requires recovery of labeled, mature protein from an ECM-enriched or spatially imaged compartment, with controls for labeling efficiency, protein assembly, and background transfer.

### 3.4 Lineage tracing and conditional genetic manipulation

Lineage tracing records the history of cells carrying a recombinase-defined label. It can reveal state changes in perivascular cells during pre-metastatic niche formation (Murgai et al., 2017). Conventional reporters do not usually tag every protein secreted by that lineage, and a labeled cell adjacent to a fiber is not proof that it produced the fiber. Long-lived ECM may also persist after the lineage disappears. Lineage history and protein history should therefore not be treated as synonymous.

Conditional deletion of an ECM gene provides causal leverage. If deletion in a defined producer reduces deposited protein and changes a tumor phenotype, it supports a requirement for that source in the tested model. In PDAC, alpha-smooth muscle actin (αSMA)-lineage Col1a1 deletion reduced fibrillar collagen and stiffness and accelerated tumor progression, whereas cancer-cell-selective collagen manipulations produced different phenotypes (Chen et al., 2021; Tian et al., 2021). Such studies provide substantially stronger evidence than transcriptional attribution. However, they are not complete provenance experiments because ECM-gene deletion can alter the producer’s adhesion, survival, migration, differentiation, stress response, and broader co-secretome. Recombination may also be incomplete or broader than the intended state. Protein deposition, producer abundance, and producer state must therefore be measured after deletion.

A conditional phenotype also cannot establish intrinsic non-equivalence. If one source is deleted while another remains in a different spatial compartment, the phenotype may reflect location or amount. The next steps are same-source rescue, cross-source rescue, abundance matching, and mechanistic comparison. Genetic epistasis downstream of a receptor is informative, but it does not substitute for replacing the missing matrix product.

### 3.5 Spatial proteomics, imaging, and structural analysis

Multiplexed immunofluorescence, imaging mass spectrometry, spatial extracellular proteomics, and ECM-targeted mass-spectrometric imaging can map proteins to microregions obscured by bulk proteomics (Macdonald et al., 2024; Boyle et al., 2020; Rujchanarong et al., 2021). Antibodies that recognize splice variants or neoepitopes can add proteoform information, although their specificity must be demonstrated in the relevant fixation and tissue context. Imaging mass spectrometry avoids antibody dependence but may sacrifice molecular completeness or cellular resolution.

Second-harmonic generation (SHG) and related nonlinear microscopy visualize fibrillar collagen without an exogenous label, including in living tumors (Bakker et al., 2022). SHG studies have defined collagen reorganization at tumor–stroma interfaces, prognostic fiber alignment, and local stiffening associated with collective invasion (Provenzano et al., 2006; Conklin et al., 2011; Koorman et al., 2022). Super-resolution imaging, electron microscopy, atomic-force microscopy, rheology, and tension probes further characterize fiber diameter, pore size, ligand exposure, stiffness, and viscoelasticity. These measurements define the final matrix state but do not identify the original producer. Fluorescence colocalization between a cell marker and a fiber is also insufficient because secreted proteins can move and persist.

This distinction has practical consequences. Tumor-cell DDR1 can promote collagen-fiber alignment and immune exclusion (Sun et al., 2021), establishing an organizer role without identifying the collagen-producing cell. CAF discoidin domain receptor 2 (DDR2) and collagen density affect stiffness, metastasis, and T-cell behavior through organization and signaling (Bayer et al., 2019; Kuczek et al., 2019). A collagen-rich image is therefore not a source map, and increased collagen content does not specify a particular fiber geometry or mechanical state.

### 3.6 Decellularized ECM as a functional comparison platform for add-back and source exchange

Decellularized matrices preserve complex cell-produced material while removing living producers. They are therefore best viewed as functional comparison platforms for testing how a pre-existing matrix state affects responder cells, rather than as direct source-attribution methods. Native or tumor-derived decellularized ECM usually integrates products from multiple cell types and retains differences in composition, architecture, stiffness, viscoelasticity, bound factors, and preparation history. Studies using primary liver cancer ECM, ovarian metastatic ECM, and decellularized organ matrices illustrate the value of this approach for preserving biologically relevant matrix cues (van Tienderen et al., 2023; Puttock et al., 2023; Pospelov et al., 2023). However, a phenotype observed after reseeding cannot be assigned to one producer or one protein unless source composition and the physical matrix state are independently controlled. Detergents, enzymes, osmotic treatments, and agitation can remove glycosaminoglycans and soluble factors, expose cryptic epitopes, disrupt basement membranes, alter fiber architecture and mechanics, or leave residual DNA and reagents (Crapo et al., 2011). If matrices from two sources require different processing or start from different cell densities, observed phenotypes may reflect technical or biomechanical differences rather than provenance.

An ideal source comparison combines source-specific knockout with direct verification that deposited target protein decreases. Same-source rescue tests whether replacing the product reverses the phenotype, whereas cross-source rescue asks whether the alternative producer’s product can substitute. Both rescues should match deposited abundance, spatial microregion, timing, fiber architecture, and bulk mechanics. Otherwise, failure to rescue cannot be attributed to producer identity. Reciprocal exchange is stronger than a one-way test, and purified-protein add-back is informative only if the relevant native proteoform and assembly state are restored.

Decellularized ECM is therefore most informative for provenance questions when combined with defined producer systems, source-specific perturbation, or matched add-back and exchange experiments, with composition and mechanics measured in parallel.

### 3.7 Evidence dimensions and causal standards

Evidence should be reported as a profile rather than a single prestige hierarchy. Transcriptional attribution is strong for cell state and timing but weak for deposition. Species-resolved proteomics is strong for protein source but weak for host-cell identity. Conditional genetics can provide strong source-specific causality while remaining weak on mechanism. Imaging is strong for architecture but weak for producer history. Table 2 makes these differences explicit across five evidence dimensions.

**TABLE 2:** Evidence-capability matrix for resolving ECM production.

Method	Source attribution	Deposited-protein evidence	Spatial information	Temporal information	Causal capacity
Single-cell RNA sequencing	High for candidate state	None	Low	Intermediate	Low
Spatial transcriptomics	Intermediate–High for candidate state	None	High for RNA	Low	Low
ECM-enrichment proteomics	Low	High	Low	Intermediate across cohorts	Low
Species-resolved xenograft proteomics	Intermediate: tumor versus host	High	Low–Intermediate	Intermediate	Low–Intermediate
Cell-selective metabolic labeling	High for labeled lineage	Intermediate–High after ECM recovery	Low–Intermediate	High: pulse window	Intermediate
Cell-selective secretome tagging	High	Intermediate for secretion; Low for stable deposition	Low	High: pulse window	Intermediate
Proximity labeling	Intermediate: proximity, not synthesis	Intermediate	Intermediate–High	High: pulse window	Intermediate
Lineage tracing	High for cell history	None unless protein is tagged	High for cells	High for cell history	Low
Conditional ECM-gene deletion	High, driver-dependent	Intermediate if deposition measured	Intermediate	High if inducible	High for producer-specific necessity
Multiplex protein imaging	Low–Intermediate	High	High	Low	Low–Intermediate
Imaging mass spectrometry/spatial proteomics	Low–Intermediate	High	Intermediate–High	Low	Low
SHG/electron microscopy/super-resolution imaging	None	Structural only	High	Intermediate if intravital	Intermediate with perturbation
Atomic-force microscopy/rheology/tension probes	None	None	Low–Intermediate	Low	Intermediate for mechanics
Decellularized ECM and reseeding	Low–Intermediate	High after validation	Intermediate	Fixed production history	Intermediate
Same-source rescue	High	High if verified	Model-dependent	Model-dependent	High for target mediation
Reciprocal cross-source exchange	High	High	High if matched	High if controlled	Highest for non-equivalence

A strong provenance claim should answer six questions. First, which cell produced and secreted the target product? Second, was that product directly detected in deposited ECM? Third, when and where was it produced and deposited? Fourth, does source-specific intervention alter deposition and phenotype without materially changing the producer population or state? Fifth, can a matched same-source product rescue the phenotype? Sixth, can a product from another source rescue under matched molecular, spatial, and mechanical conditions? If not, what proteoform, assembly, or receptor mechanism explains the failure? Most current studies answer the first three; fewer reach the fourth, and very few complete the fifth and sixth. Table 3 summarizes minimum experimental standards for progressively stronger provenance claims, while Figure 2 integrates the evidence dimensions and causal workflow.

**TABLE 3:** Minimum experimental standards for causal provenance claims.

Claim	Minimum evidence	Recommended controls	Common false inference	Decisive experiment
Cell X expresses ECM gene Y	Cell-resolved RNA/reporting	Ambient RNA; *in situ* RNA	X deposited Y	Cell-selective nascent Y plus deposited-protein detection
Cell X secretes Y	Cell-selective label/tag or species peptide	Label efficiency; soluble versus intracellular fractions	Secreted Y forms stable ECM	Recover mature labeled Y from ECM-enriched tissue
Cell X deposits Y	Source-resolved mature protein in ECM	Soluble depletion; extraction and spatial controls	Adjacent cell produced the fiber	Pulse–chase source label co-registered with deposit
X is required for Y deposition	X-specific Y loss plus quantitative deposited-protein reduction	Recombination; producer number/state; other sources	Phenotype is caused only by Y loss	Same-source rescue of deposition and phenotype
X-derived Y affects tumor phenotype	Source intervention, deposition, phenotype	Cell-autonomous effects; co-secretome	Y from X is intrinsically special	Same-source rescue, then cross-source rescue
X- and Z-derived Y are non-equivalent	Reciprocal source exchange with product specified	Match abundance, timing, site, architecture, mechanics, receptors	Two conditional knockouts equal a source swap	Reciprocal *in vivo* deposition in the same niche
Source-linked proteoform mediates function	Proteoform-resolved analysis and perturbation	Purity; total Y; assembly; protease sensitivity	Same gene symbol means same molecule	Proteoform rescue plus receptor epistasis
Therapy causes source replacement	Longitudinal producer map plus source-resolved deposited Y	Legacy ECM; injury; systemic sources	Recovered total Y came from original source	Block replacement producer and test recovery
Matrix legacy persists	Producer pulse/ablation plus persistence of labeled deposit	New synthesis; half-life; treatment injury	Current cells made current ECM	Pulse–chase through producer loss and recurrence

**FIGURE 2:**
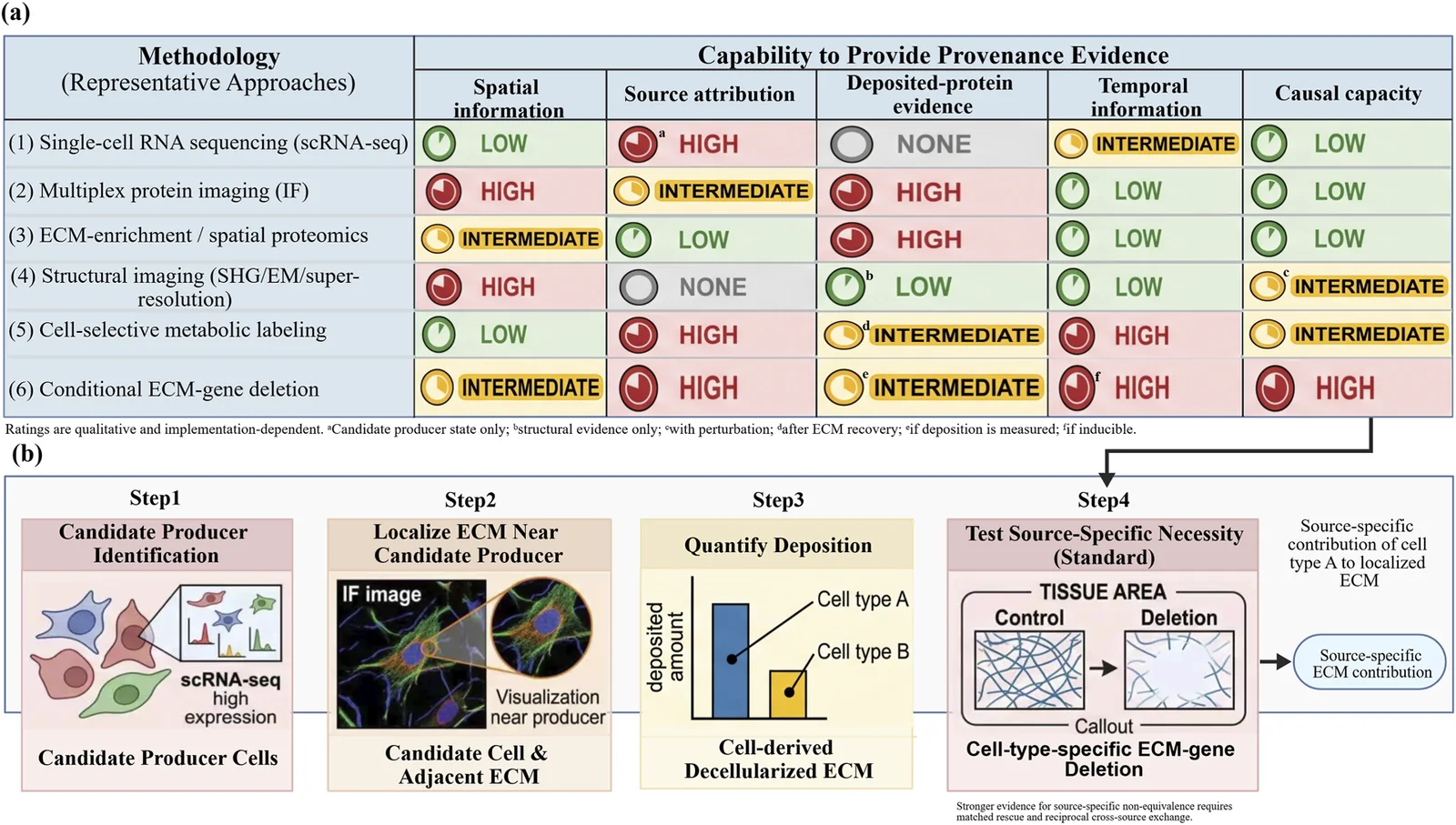
Evidence dimensions and minimum causal standards for testing ECM provenance. **(a)** Qualitative evidence-capability matrix comparing representative approaches for spatial information, source attribution, deposited-protein evidence, temporal information, and causal capacity. Ratings are implementation-dependent, and superscript letters indicate method-specific qualifications shown beneath the matrix. **(b)** Stepwise workflow for testing source-specific extracellular matrix (ECM) contribution, comprising candidate producer identification, localization of ECM adjacent to the candidate producer, quantification of deposited ECM, and assessment of source-specific necessity by cell-type-specific ECM-gene deletion. This workflow establishes source-specific contribution, whereas stronger evidence for source-specific functional non-equivalence requires matched rescue and reciprocal cross-source exchange. scRNA-seq, single-cell RNA sequencing; IF, immunofluorescence; SHG, second-harmonic generation; EM, electron microscopy. Created with BioGDP.com (Jiang et al., 2025).

## 4 Molecular and structural mediators

### 4.1 Alternative splicing and proteoforms

Different producer states can use different promoters and splice programs. Cellular fibronectin commonly includes extra domains A and B (EDA and EDB), whereas plasma fibronectin lacks these segments. EDA-containing fibronectin has been localized to tumor vasculature and genetically interrogated in angiogenesis models (Rybak et al., 2007; Astrof et al., 2004). Osteoblast-derived EDA-fibronectin can shape antitumor immunity in bone, and CAF-associated EDA programs have been linked to therapy resistance (Rossnagl et al., 2016; Dong et al., 2024). Recent splice manipulation further indicates that an FN1 isoform can create an invasion-permissive matrix (Franco-Valls et al., 2023). Together, these studies establish that transcript identity can matter. They support provenance dependence only when a producer state is shown to generate the isoform and when isoform, rather than producer abundance or location, explains the phenotype.

Tenascin C also has multiple splice forms, and laminin function depends on chain combinations. Expression of TNC or a laminin gene in two cell types therefore does not establish identical products. The relevant comparison should proceed from gene identity to transcript, proteoform, and supramolecular state. Source-linked splicing is a plausible determinant mechanism, but cell-resolved isoform deposition remains poorly resolved for most tumor ECM proteins.

### 4.2 Modification and proteolysis

Producer cells differ in glycosyltransferases, prolyl and lysyl hydroxylases, sulfation enzymes, chaperones, and redox systems. These differences can alter secretion, fibril stability, receptor binding, and protease susceptibility. P4HA1-dependent collagen prolyl hydroxylation has been linked to HIF-1α stabilization and TNBC chemoresistance (Xiong et al., 2018). PLOD2-linked succinate metabolism contributes to epithelial–mesenchymal plasticity and cancer cell stemness (Tong et al., 2023). HSP47-dependent collagen handling has been linked to metastatic progression (Wang et al., 2024; Ling et al., 2026), while proteome-wide analyses document altered proline hydroxylation in PDAC (Wu et al., 2025). Cell-autonomous prostate cancer and CAF collagen-processing studies further show that COL1A1 abundance alone does not define collagen state (Ippolito et al., 2024; Cencioni et al., 2025). Citrullination differences between breast tumors and adjacent tissue provide another modification landscape whose producer attribution remains unresolved (Tillotson et al., 2023).

Post-secretory proteolysis can override source. Endostatin and tumstatin, generated from collagens XVIII and IV, have receptor activities distinct from their parent networks (O'Reilly et al., 1997; Sudhakar et al., 2003). Collagen cleavage can expose DDR1-readable states, and proteases can release matrikines whose effects are not predictable from intact-protein abundance (Su et al., 2022). Evidence that modification and cleavage alter ECM function is strong, but evidence that two producer types create different deposited modifications under abundance- and space-matched conditions remains much rarer.

### 4.3 Secretion and co-secretomes

The producer’s endoplasmic-reticulum capacity, chaperones, glycosylation machinery, proteases, inhibitors, and vesicle pathways determine what is secreted and what accompanies it. HSP47-dependent collagen handling by tumor cells or in tumor–platelet interactions shows that chaperone programs can alter collagen secretion and downstream behavior (Xiong et al., 2020; Wang et al., 2024). Nicotinamide N-methyltransferase (NNMT)-driven CAF metabolic remodeling alters a broader stromal program rather than a single ECM protein (Eckert et al., 2019). Extracellular-vesicle-associated LOX and exosomal tenascin C further show that secretion route can alter localization and molecular context (Liu et al., 2023; Mirzaei et al., 2018).

This mechanism is plausible but experimentally difficult to isolate. Deleting a single ECM gene may rewire the broader secretory state, while conditioned medium transfers multiple factors. A phenotype attributed to “fibroblast-derived protein X″ could instead reflect protease balance, growth factors, vesicles, or assembly partners. Matched recombinant-protein rescue can test molecular sufficiency but may fail because it omits the native co-secretome. Conversely, rescue with whole conditioned medium cannot isolate the target. Fractionation, proteoform-resolved analysis, and receptor epistasis are therefore needed to narrow causality.

### 4.4 Supramolecular assembly

Source-linked co-secretion can shape stoichiometry, fiber diameter, pore size, network continuity, and growth-factor retention. Fibronectin is particularly instructive because endothelial, fibroblast, tumor-cell, and plasma sources may enter different assembly routes. CD93/MMRN2 perturbation disrupts fibrillogenesis without lowering FN1 transcription (Lugano et al., 2018), while an engineered non-fibrillar or arginine-glycine-aspartate (RGD)-deficient fibronectin state can alter metastatic behavior (Urtreger et al., 1998). Inhibiting fibronectin accumulation can change tumor growth, but such interventions target the final network rather than a single producer (Ghura et al., 2021).

SNED1 provides an emerging example of matrix assembly. Recent work indicates that fibrillar SNED1 assembly requires fibronectin and collagen I, while motif perturbation changes architecture (Leverton et al., 2026; Pally et al., 2026). Unless direct binding is demonstrated, these observations support dependence, colocalization, or candidate co-assembly rather than a defined binding interaction. They also do not establish that SNED1 produced by two cell types would be functionally non-equivalent.

### 4.5 Spatial mechanics and receptor reading

The same proteoform can have different effects at the tumor core, invasive front, vessel wall, immune-exclusion boundary, nerve, or premetastatic niche because local receptors, forces, proteases, and responder cells differ. Nerve-associated ECM and perivascular tenascin C illustrate this context dependence (Di Chiaro et al., 2024; Hongu et al., 2022). Crosslinking inhibition can improve T-cell migration and checkpoint response (Nicolas-Boluda et al., 2021). Matrix viscoelasticity can also promote liver cancer before overt cirrhotic stiffness develops (Fan et al., 2024).

A related example comes from our recent study of lung adenocarcinoma, which integrated single-cell and spatial transcriptomic data with ligand–receptor communication analysis and identified a potential SLC2A1–CAF–collagen signaling axis. POSTN + CAFs showed the strongest inferred contribution to collagen-mediated signaling toward SLC2A1+ malignant epithelial cells (Song et al., 2026). Although this study did not directly resolve collagen provenance or matrix mechanics, it illustrates how a malignant-cell state can be linked to a specific stromal collagen-signaling context.

These findings show that final matrix state and receptor context can explain an apparent producer effect. If fibroblast collagen is peritumoral and cancer-cell collagen is pericellular, a phenotype difference may arise from micrometer-scale location. If one niche expresses integrin α3β1 and another expresses leukocyte-associated immunoglobulin-like receptor 1 (LAIR1) or DDR1, the same material may be read differently. Producer identity is causal only insofar as it generates a persistent, measurable mediator state. Once molecular, spatial, mechanical, and receptor-readable states are genuinely matched, residual differences should prompt a search for an unmeasured mediator rather than attribution to provenance alone.

## 5 Rewiring ECM production during cancer progression

### 5.1 Early lesions

Tumorigenesis does not simply amplify a fixed matrix program. It changes which cells synthesize particular products, the states those cells occupy, and when and where their products are deposited. Resident fibroblasts, epithelial or malignant cells, endothelial cells, pericytes, macrophages, and recruited mesenchymal populations can enter or leave the producer pool. Temporal breast and pancreatic matrisome studies show staged protein changes rather than a single monotonic desmoplastic response (Papanicolaou et al., 2022; Pereira et al., 2024). Spatial studies likewise identify regional ECM competence for tumor initiation and progressive stromal changes during invasion (Risom et al., 2022; Bansaccal et al., 2023).

This process constitutes rewiring of ECM production. Producer identity or state may change, and production or deposition context may change even when the dominant producer does not. Early malignant cells can acquire a cell-autonomous ECM program while resident fibroblasts transition into contractile, inflammatory, or stress-associated states. In other settings, a pre-existing organ matrix determines which transformed cells can expand. Bulk tumor-versus-normal measurements cannot distinguish these routes. Longitudinal cell-state maps, pulse labeling, and deposited-protein measurements are needed to determine whether a new producer has emerged or an existing matrix is being remodeled.

### 5.2 Tumor core and invasive front

At an invasive edge, three questions should be separated: who supplies the major proteins, who organizes the tracks, and where invasive cells read them. CAF-led studies support fibroblast fibronectin assembly (Attieh et al., 2017), whereas tumor cells can organize autocrine matrices or produce their own collagen (Tian et al., 2021; Shinde et al., 2018). Collagen alignment at the tumor boundary can predict aggressive behavior even when producer identity is unknown (Provenzano et al., 2006; Conklin et al., 2011). In some tumors, deposition context and architecture may therefore explain more than producer identity.

Tumor-cell ECM is not uniformly pro-invasive. Autocrine fibronectin restrained breast-cancer metastasis (Shinde et al., 2018). By contrast, tumor-cell collagen I homotrimers promoted PDAC (Chen et al., 2022), while collagen III supported dormancy or tumor restriction in other settings (Di Martino et al., 2022; Stewart et al., 2024). These studies involve different products, proteoforms, stages, and endpoints and therefore do not support a general rule for “tumor-derived matrix.” The invasive front also illustrates role separation. CAFs may secrete collagen, tumor cells may align existing fibers through DDR1 signaling, and macrophage- or fibroblast-derived LOX-family enzymes may crosslink the network. SHG can define the resulting architecture, but a producer claim requires a cell-selective protein label or allele.

### 5.3 Perivascular niches and intravasation

Tumor cells, CAFs, endothelial cells, and pericytes converge at blood vessels. Endothelial CD93–MMRN2 signaling organizes perivascular fibronectin fibrils, and endothelial fibronectin can support tumor-cell adhesion in defined models (Lugano et al., 2018; Van Passen et al., 2025). However, endothelial fibronectin or its canonical receptors were dispensable for tumor angiogenesis in other conditional models (Murphy et al., 2015). This negative result is mechanistically informative because plasma fibronectin, stromal production, or alternative matrices may compensate. A universal requirement for the endothelial source is therefore untenable.

Perivascular CAF stress programs can drive angiogenesis, and tumor–stroma boundary organization can influence therapeutic response (Verginadis et al., 2022; Feng et al., 2024). Cells that synthesize a vascular basement-membrane chain need not be the same cells that assemble it or proteolyze it during intravasation. Perivascular staining therefore does not establish endothelial synthesis. A decisive design would separately pulse-label nascent protein in endothelial and perivascular lineages, image mature deposition over time, and perturb assembly or proteolysis independently.

### 5.4 Premetastatic source relay

Premetastatic niches make the distinction between signaling source and matrix producer especially clear. Primary tumor cells can send exosomes or cytokines, whereas resident cells in the liver, lung, bone, or omentum synthesize local ECM. Pancreatic-cancer exosomes initiate a liver cascade involving Kupffer and stellate cells and a fibronectin-rich niche (Costa-Silva et al., 2015). A widely cited report linked exosomal integrins to organotropic targeting, although the article now carries an expression of concern (Hoshino et al., 2015; Hoshino et al., 2026). Melanoma exosomes have also been reported to educate bone-marrow progenitors (Peinado et al., 2012). These studies identify upstream relay signals but do not assign production of the distant matrix to the tumor cell.

Perivascular-cell plasticity can contribute to pre-metastatic niche formation (Murgai et al., 2017), whereas a collagen-rich omentum can support integrin α2-mediated peritoneal metastasis (Huang et al., 2020). Other niche-remodeling routes include diet- and platelet-associated effects (Hergueta-Redondo et al., 2025), chronic-stress-associated neutrophil remodeling (He et al., 2024), and hypoxia-induced LOX-dependent bone-marrow-cell recruitment (Erler et al., 2009). The classical vascular endothelial growth factor receptor 1 (VEGFR1)-positive-cell/fibronectin-rich niche is compatible with several producer assignments and cannot be reduced retrospectively to one source without protein-level evidence (Kaplan et al., 2005). Periostin is better viewed within this relay framework than as a separate provenance case. Host periostin supports metastatic colonization (Malanchi et al., 2012; Fukuda et al., 2015; Wang et al., 2016; Gillot et al., 2022; Null et al., 2023), whereas tumor-cell periostin has been linked to a cytokine program that maintains breast cancer stemness (Lambert et al., 2016). Together, these studies support stage- and organ-dependent source convergence rather than cross-source non-equivalence.

### 5.5 Extravasation dormancy and recurrence

Disseminated cells can exploit an existing matrix, remodel it, or synthesize a new pericellular niche. Tumor-cell tenascin C supports early lung colonization, whereas S100 calcium-binding protein A4 (S100A4)-positive stromal cells provide tenascin C during metastatic outgrowth (Oskarsson et al., 2011; O'Connell et al., 2011). This sequence suggests source relay or replacement but does not establish intrinsic differences between tumor- and stromal-derived proteins. Section 6.2 examines the comparative evidence.

Dormancy adds temporal ambiguity. Perivascular and cell-matrix niches can regulate quiescence, survival, and recurrence (Ghajar et al., 2013; Ohta et al., 2022; Sreekumar et al., 2024). Tumor-cell-organized fibronectin and collagen III have been implicated in dormant states (Di Martino et al., 2022; Barney et al., 2020). During reactivation, an ECM program may be newly synthesized, inherited, or exposed by inflammation. Chemotherapy-associated neutrophil extracellular traps can awaken dormant cells through matrix-remodeling proteases, illustrating a post-secretory trigger rather than a change in the original producer (He et al., 2025).

### 5.6 Therapy-induced replacement and legacy

Chemotherapy, radiotherapy, antiangiogenic therapy, immunotherapy, and stromal interventions alter the survival and state of candidate producers. Radiotherapy can reorganize brain-tumor microenvironments (Watson et al., 2024), while collagen I–CXCL1 signaling can contribute to radioresistance in esophageal cancer (Yang et al., 2023). TNC has also been implicated in radiotherapy responses in head-and-neck and oral squamous carcinoma models (Loustau et al., 2026; Spenlé et al., 2021). TGF-β-associated immune exclusion, CAF states, and tumor-stroma boundaries influence checkpoint response (Feng et al., 2024; Mariathasan et al., 2018; Kieffer et al., 2020). Together, these studies support treatment-associated rewiring, but a post-treatment cell map cannot retrospectively identify which cells deposited persistent ECM.

Source replacement should be anticipated when one producer is inhibited. A surviving fibroblast state, macrophage, endothelial cell, malignant subclone, or systemic source may restore the same gene-identity product. Recent matrix- and fibrosis-directed interventions provide opportunities to measure such compensation (Tang et al., 2025; Khan et al., 2025; Parikainen et al., 2026; Wang et al., 2025). The restored material may occupy a different microregion or have a different proteoform. Post-treatment analysis should identify replacement producers, quantify deposited protein, map its architecture and location, and test whether the replacement matrix restores the phenotype.

Matrix legacy can persist after source loss. Treatment may clear malignant producers while leaving crosslinked collagen, basement membranes, matrix-bound vesicles, and mechanically stable tracks. Tissue injury can likewise generate material that outlives the acute inflammatory population. Producer-directed and matrix-state-directed interventions therefore operate on different timescales. Early failure of producer inhibition may reflect legacy matrix rather than absence of producer-mediated biology. Figure 3 summarizes these temporal changes in ECM provenance.

**FIGURE 3:**
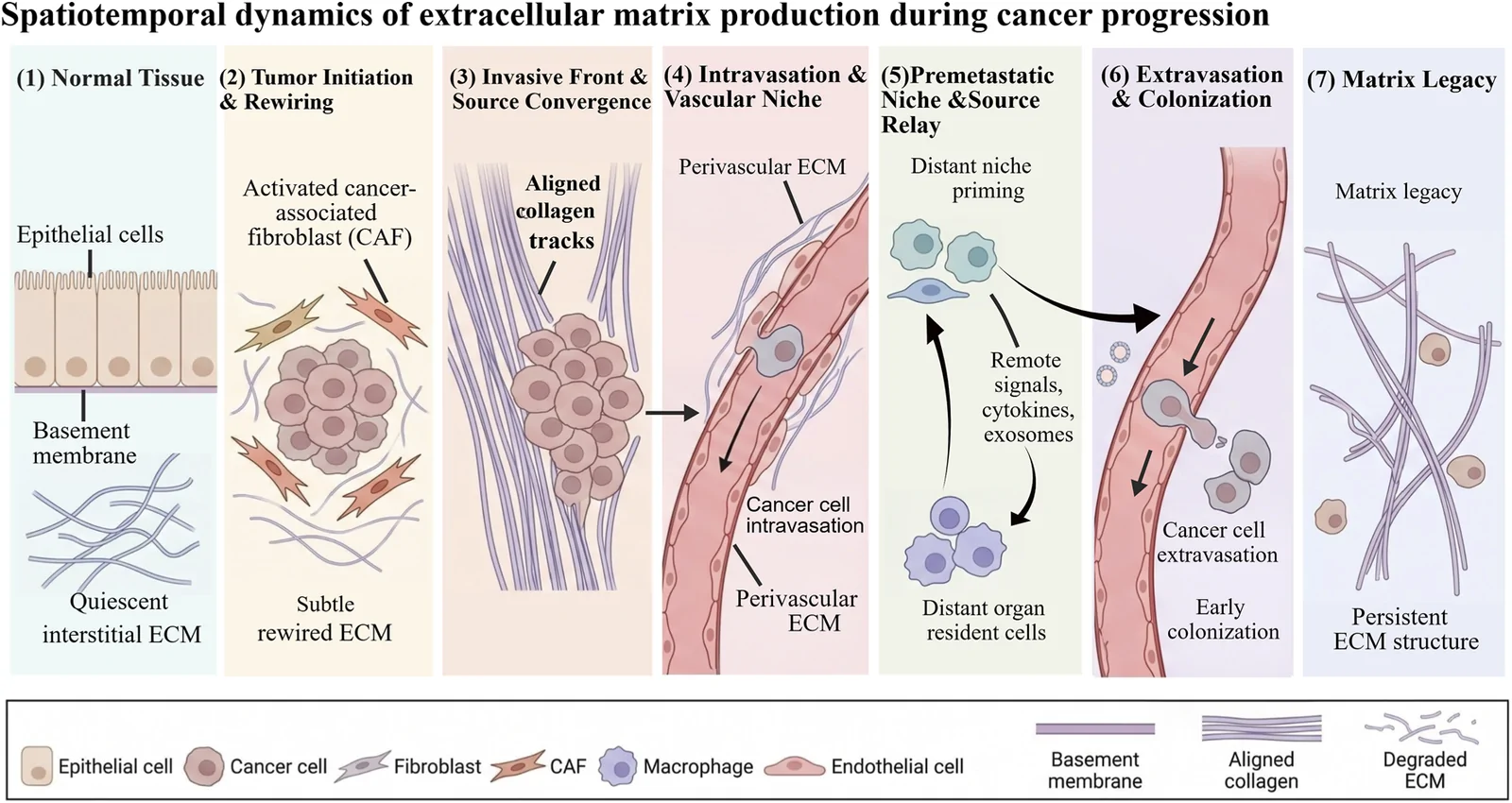
Rewiring, relay, replacement, convergence, and legacy during cancer progression. Schematic representation of extracellular matrix (ECM) changes from normal tissue through tumor initiation and rewiring, invasive-front source convergence, intravasation and vascular-niche remodeling, premetastatic niche formation and source relay, extravasation and colonization, and matrix legacy. The model illustrates changes in cellular contributors and deposited matrix architecture, including cancer-associated fibroblast activation, aligned collagen tracks, perivascular ECM, distant niche priming by remote signals, cytokines and exosomes, and persistence of ECM structures after changes in the producing cell population. CAF, cancer-associated fibroblast. Created with BioGDP.com (Jiang et al., 2025).

## 6 Comparative case studies

### 6.1 Collagen I and proteoform identity

αSMA-positive myofibroblasts supply much of the fibrillar collagen in PDAC, but malignant cells also produce a small, biologically consequential fraction. Species-specific peptide analysis, ECM enrichment, lineage-directed alleles, SHG, stiffness measurements, and procollagen-processing assays have been used to separate these contributions (Chen et al., 2022; Chen et al., 2021; Tian et al., 2021). These designs go beyond RNA attribution, although none provides a fully matched reciprocal *in vivo* source exchange.

Deleting Col1a1 in αSMA-positive myofibroblasts reduced fibrillar collagen and stiffness yet accelerated PDAC, accompanied by immunosuppressive myeloid changes (Chen et al., 2021). A related model supported tumor-restrictive type I collagen despite tumor-promoting effects of the broader fibroblast compartment (Bhattacharjee et al., 2021). Malignant-cell-selective studies produced apparently discordant outcomes. Bone morphogenetic protein 1 (BMP1)-dependent processing and deposition of tumor-cell collagen restrained PDAC in one study (Tian et al., 2021), whereas cancer-cell collagen I α1 homotrimers promoted growth and immune evasion in another (Chen et al., 2022). Differences in model, processing, proteoform, and endpoint preclude a simple “fibroblast good, tumor bad” interpretation.

Proteoform-resolved rescue in the homotrimer study most closely approaches a producer-mediated determinant model. PDAC cells suppressed collagen type I alpha 2 chain (COL1A2) and produced α1 homotrimers, whereas fibroblasts produced conventional α1/α2 heterotrimers. Cancer-cell Col1a1 deletion delayed disease despite maintained or increased stromal collagen. Purified homotrimer rescued spheroid behavior more effectively than heterotrimer and activated α3β1-integrin, focal adhesion kinase (FAK), protein kinase B (AKT), and extracellular signal-regulated kinase (ERK) signaling (Chen et al., 2022). These data support functional non-equivalence between distinct proteoforms generated by different producer programs, not an independent record of producer identity in chemically and structurally identical collagen.

Total abundance, homotrimer fraction, modification, cleavage, crosslinking, alignment, and receptor expression remain alternative explanations. HSP47-dependent folding and LOX-mediated crosslinking can alter collagen maturation and organization (Wang et al., 2024; Saatci et al., 2020). LAIR1 can read collagen-associated immune states (Peng et al., 2020), while postpartum remodeling shows that time and space can dominate a collagen phenotype (Lyons et al., 2011).

The collagen literature therefore provides the strongest current example of a producer-mediated effect acting through proteoform identity. Fibroblast- and tumor-cell collagen programs can be required for distinct PDAC phenotypes, and α1 homotrimers and α1/α2 heterotrimers are functionally different. Whether collagen remains non-equivalent after abundance, location, architecture, modification, and receptor access are matched is unknown. Resolving this question requires reciprocal *in vivo* deposition of characterized proteoforms in the same microregion with matched mechanics and receptor availability.

### 6.2 Tenascin C and stage-dependent replacement

Breast cancer cells can synthesize TNC during early lung colonization, whereas S100A4-positive host stromal cells provide it during later metastatic outgrowth (Oskarsson et al., 2011; O'Connell et al., 2011). Perivascular or reprogrammed stromal cells contribute in other models (Hongu et al., 2022; Lee et al., 2022). Brain tumor-initiating cells can export exosome-associated TNC that suppresses T-cell activity (Mirzaei et al., 2018). TNC can also inhibit T-cell activation and protect cancer stem-like cells from immune surveillance (Jachetti et al., 2015). The evidence therefore supports multiple producers and stage-dependent replacement, while producer identities remain coupled to different locations, splice forms, and secretory environments.

Deligne and colleagues directly compared host- and tumor-derived TNC in syngeneic mouse tumor models. Host-derived TNC was associated with antitumoral macrophages, whereas tumor-derived TNC favored an immunosuppressive phenotype. TNC- or toll-like receptor 4 (TLR4)-targeting antibodies altered these states and reduced tumor growth and spread (Deligne et al., 2020). This provides unusually direct source-stratified immune evidence. However, the study did not exchange equal amounts of isoform-matched TNC between identical spatial compartments, and the manipulations could also alter tumor or host co-secretomes.

Taken together, the Oskarsson and O'Connell studies are compatible with a relay in which early malignant TNC production is replaced or supplemented by host production (Oskarsson et al., 2011; O'Connell et al., 2011). They do not compare the two products under matched conditions. Alternative explanations include temporal availability, perivascular versus pericellular deposition, splice variants, glycosylation, vesicle association, macrophage composition, and local TLR4 accessibility. Radiotherapy-associated tenascin C phenotypes further show that treatment can alter both production context and biological reading (Loustau et al., 2026; Spenlé et al., 2021).

The tenascin C evidence is best classified as source-associated immune biology with stage-dependent replacement. Tumor and host cells can both supply consequential tenascin C, and Deligne et al. reported opposing macrophage phenotypes in mouse models. Whether this difference persists after abundance, isoform, spatial deposition, and co-secretome are matched remains unresolved. A decisive reciprocal test would express the same characterized proteoform at the same site and level before macrophage and tumor assays.

### 6.3 Fibronectin and source redundancy

Fibronectin arises from several local and systemic compartments and is therefore particularly susceptible to source redundancy. Tumor-cell and plasma fibronectin have been manipulated directly (Malik et al., 2010; Shinde et al., 2018), while endothelial pathways can control fibril assembly (Lugano et al., 2018). This diversity makes fibronectin a stringent test of apparent source dependence and functional interchangeability.

Endothelial CD93 deficiency disrupted perivascular fibronectin fibrils without lowering FN1 transcription (Lugano et al., 2018), whereas CAFs align invasion-guiding tracks (Attieh et al., 2017). Tumor-cell-organized fibronectin has also been linked to dormancy (Barney et al., 2020). Plasma and cellular fibronectin differ in EDA/EDB splicing. Genetic deletion studies indicate that individual EIIIA or EIIIB segments can be dispensable for angiogenesis in some contexts (Astrof et al., 2004), while more recent splice manipulation supports isoform-specific effects on invasion-permissive matrix formation (Franco-Valls et al., 2023). These experiments compare molecular variants or assembly states rather than producer identity alone. Soluble, adsorbed, and fibrillar fibronectin likewise represent distinct receptor-readable states.

Plasma fibronectin promoted lung metastasis (Malik et al., 2010), whereas autocrine tumor-cell fibronectin restrained breast-cancer metastasis (Shinde et al., 2018). Endothelial assembly promoted glioma vascular organization (Lugano et al., 2018), but conditional loss of endothelial fibronectin or cognate integrins did not prevent angiogenesis in other models (Murphy et al., 2015). Macrophage-derived fibronectin can suppress antitumor immunity (Li et al., 2026), while fibronectin from other stromal populations can also modulate antitumor immune responses (Lubosch et al., 2025). These findings remain compatible with both provenance-associated and provenance-independent explanations: another source may replace the protein, or a shared fibronectin state may function equivalently once deposited.

Local and systemic fibronectin sources therefore contribute to distinct tumor phenotypes, while compensation and assembly differences remain plausible explanations for apparent source dependence. Evidence is strongest when lineage-specific perturbation is combined with deposited-fiber imaging, splice analysis, or systemic-source genetics. A reciprocal, compensation-aware source exchange that matches proteoform, abundance, location, assembly, mechanics, and receptor readouts remains lacking.

### 6.4 Emerging tumor-cell-derived matrix organizers: SNED1 and LTBP3

#### 6.4.1 Direct evidence

Species-resolved ECM proteomics in human breast-cancer xenografts assigned SNED1 and LTBP3 peptides to the human tumor compartment and detected them preferentially in highly metastatic tumors (Naba et al., 2014). Tumor-cell shRNA against either gene reduced invasion and metastatic dissemination without a comparable reduction in primary-tumor growth in the tested system. Matrix architecture also changed, including tumor encapsulation and peripheral collagen accumulation. Follow-up work supported a role for LTBP3 in early metastatic dissemination (Deryugina et al., 2018). Together, these findings provide coarse protein-level source attribution and source-specific perturbation evidence.

Recent biochemical and cell-based studies have refined the mechanism of SNED1. SNED1 supports adhesion through RGD-binding integrins, and its fibrillar assembly depends on fibronectin and collagen I in tested matrix systems (Leverton et al., 2026; Pally et al., 2025). Mutation of a leucine-aspartate-valine (LDV) motif alters matrix architecture and cell proliferation (Pally et al., 2026). Domain analysis and predicted interaction networks provide additional hypotheses (Vallet et al., 2021).

#### 6.4.2 Suggestive evidence

Colocalization or assembly dependence suggests that SNED1 participates in fibronectin- and collagen-containing networks. It does not establish direct SNED1–fibronectin binding or identify which cell supplies each partner *in vivo*. Likewise, an invasion or metastasis phenotype after tumor-cell knockdown is consistent with a tumor-cell matrix-organizer function but may also reflect altered tumor-cell state, adhesion, TGF-β handling, or co-secretome.

#### 6.4.3 Unresolved

Neither SNED1 nor LTBP3 has undergone a protein-matched same-source rescue followed by reciprocal exchange between tumor and stromal producers *in vivo*. It remains unknown whether another producer can deposit a molecularly matched product at the same location or whether source-linked modifications exist. The functional equivalence of the resulting matrices is therefore unresolved. For SNED1, direct binding partners in native tumor ECM and the receptor consequences of specific assembled states require further validation. For LTBP3, structural ECM function must be separated from latent TGF-β regulation.

Evidence for SNED1 and LTBP3 therefore supports tumor-compartment protein assignment and tumor-cell-specific necessity in a breast-cancer xenograft model, with emerging receptor and assembly mechanisms for SNED1. Producer-specific functional non-equivalence remains untested. The strongest evidence combines species-specific peptide assignment with source-directed knockdown. A deposition-verified rescue and reciprocal tumor/stromal source swap that matches proteoform, abundance, location, and TGF-β context are still required.

## 7 Alternative explanations and translational hypotheses

### 7.1 Confounders and causal inference

An apparent source-associated phenotype should first trigger a confounder audit. Total deposited protein may differ because of producer number, per-cell output, secretion efficiency, or protein half-life. Production stage and deposition microregion may also differ, as may the target proteoform, co-secretome, fiber architecture, and mechanics. Conditional manipulation can alter producer survival or state, while plasma leakage or another systemic source can contribute additional material. Long-lived historical matrix may persist. Technical confounders include species-specific assembly artifacts in xenografts and unequal damage during decellularization. Finally, responder cells may express different receptors across the compared locations.

The causal sequence should be explicit. First, verify the deposited target and match abundance, location, timing, and producer state. Next, perform same-source rescue and reciprocal cross-source rescue before assigning the phenotype to a proteoform, architectural, or receptor mechanism. Failure at any step does not make the study uninformative; it places the conclusion at an earlier evidence dimension.

Several negative or discordant findings illustrate this logic. Endothelial fibronectin can be dispensable in some angiogenesis models despite strong endothelial assembly evidence elsewhere (Lugano et al., 2018; Murphy et al., 2015). Myofibroblast collagen I restrains PDAC in a conditional model, whereas tumor-cell collagen I can restrain or promote disease depending on processing and proteoform (Chen et al., 2022; Chen et al., 2021; Tian et al., 2021). Periostin can originate from host or tumor cells (Malanchi et al., 2012; Lambert et al., 2016), and TNC production can shift from tumor cells to S100A4-positive stroma during metastatic outgrowth (Oskarsson et al., 2011; O'Connell et al., 2011). These observations argue against a universal ECM-source rule and favor hypotheses defined by model, stage, and molecular state. Figure 4 summarizes the major confounders and causal decision framework.

**FIGURE 4:**
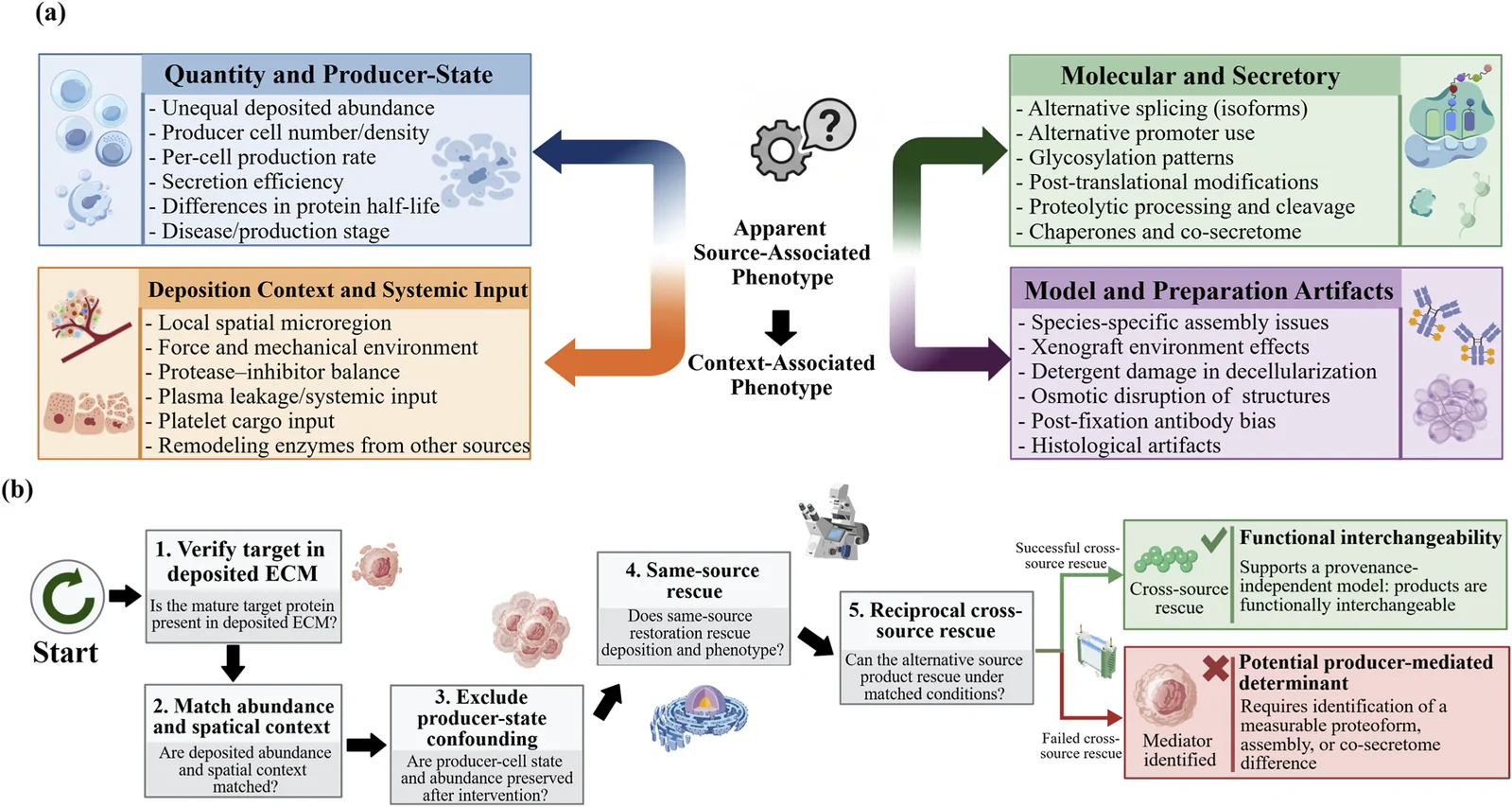
Alternative explanations and a causal decision tree for ECM provenance claims. **(a)** Confounder map for an apparent source-associated phenotype, including differences in matrix quantity and producer state, molecular and secretory properties, deposition context and systemic inputs, and model or preparation artifacts. **(b)** Causal decision tree for testing extracellular matrix (ECM) provenance. The workflow sequentially verifies the target in deposited ECM, matches abundance and spatial context, excludes producer-state confounding, and tests same-source and reciprocal cross-source rescue. Successful cross-source rescue supports functional interchangeability and a provenance-independent model, whereas failed rescue motivates identification of measurable proteoform, assembly, or co-secretome differences consistent with a potential producer-mediated determinant. Created with BioGDP.com (Jiang et al., 2025).

### 7.2 Biomarker interpretation

High FN1, COL1A1, or periostin (POSTN) can report very different processes. FN1 may indicate a malignant-cell adhesion program, CAF expansion, angiogenic endothelium, activated macrophages, tissue injury, or influx of plasma fibronectin. COL1A1 may reflect myofibroblast abundance, a tumor-cell collagen program, wound repair, crosslinked legacy matrix, or activation of a distant metastatic organ. POSTN may reflect a fibroblastic niche, a treatment-associated wound state, or a tumor-cell program. Bulk RNA and even bulk protein collapse these alternatives.

A provenance-aware biomarker should capture five attributes when feasible: predominant producing cell and state, spatial microregion, transcript or proteoform, structural state, and disease or treatment stage. Species-resolved proteomics, spatial extracellular proteomics, and integrated colorectal ECM-origin mapping show that partial versions of this profile are technically feasible (Naba et al., 2014; Macdonald et al., 2024; Lee et al., 2025). In patient samples, however, cell type cannot be inferred solely from the most correlated single-cell reference. Orthogonal protein evidence or validated cell-selective labels remain necessary.

Provenance can improve interpretation even when it is not itself causal. A fibroblast-origin collagen signal may be more specific for a stromal state than total collagen, while a tumor-origin collagen proteoform may identify a malignant biosynthetic program. Conversely, a circulating ECM fragment may primarily report remodeling rather than the producer of the parent matrix. Biomarker claims should therefore state which link in the causal chain the assay measures.

### 7.3 Therapeutic hypotheses

Resolving provenance changes what can be tested therapeutically. One strategy removes or reprograms a producer. FAP-directed cellular approaches can reduce fibrosis in PDAC models, but perturbing fibroblast-derived matrix can also remove tumor-restrictive functions (Chen et al., 2021; Wang et al., 2025). A second strategy targets cell-state-linked secretory programs, including HSP47-dependent collagen handling (Wang et al., 2024), NNMT-linked CAF metabolic remodeling (Eckert et al., 2019), and stress-induced matrix programs (Insua-Rodríguez et al., 2018). A third targets splicing, hydroxylation, proteolysis, assembly, or crosslinking. A fourth targets receptors that read a defined matrix state, such as integrin, DDR1, or LAIR1 pathways. The most rational target is the layer supported by the strongest causal evidence.

These implications are experimental design principles, not clinically validated provenance-selective interventions. Human spatial or outcome associations can support biomarker relevance but not therapeutic causality. Genetically engineered mouse models can support a producer requirement within that model. Cell culture and purified proteins can define receptor or proteoform mechanisms but may not reproduce native matrix. Crosslinking and fibronectin-matrix interventions have animal support in specified settings (Nicolas-Boluda et al., 2021; Khan et al., 2025). Such approaches should not be described as producer-selective therapies unless the affected producer and deposited product are demonstrated.

The producer-mediated determinant model predicts that targeting a source-linked proteoform or its biosynthetic enzyme may spare a functionally beneficial product from another source. The provenance-associated model predicts that targeting location, architecture, or receptor context will outperform producer ablation. The provenance-independent model predicts that another cell can replace the product, making source-directed treatment fragile. These are experimentally distinguishable hypotheses rather than claims of clinical readiness.

### 7.4 Therapeutic compensation

After a source-directed intervention, total-protein measurements alone are insufficient. Four outcomes should be distinguished. If the target remains low and the phenotype follows, the result supports source-specific necessity. If total protein recovers from a replacement producer and function also recovers, it supports interchangeability. If total protein recovers but deposition shifts and function changes, the result supports a spatial mediator. If total protein and location recover but function remains different, proteoform, co-secretome, assembly, or receptor differences should be investigated.

Therapy studies should prospectively identify substitute producers by longitudinal single-cell or spatial profiling and then verify their deposited product with proteomics, imaging, or cell-selective labeling. They should test whether total target levels recover, whether fibers or mechanics normalize, and whether the replacement product restores the relevant tumor phenotype. This design converts “stromal compensation” from a retrospective explanation into a testable source-replacement mechanism.

## 8 Discussion and perspectives

Producer identity is an upstream causal variable, but its functional effect must be mediated through persistent molecular, spatial, mechanical, or assembly differences. Published examples show why these layers must remain separate. PDAC collagen I links producer programs to proteoform-specific integrin signaling, whereas collagen alignment, stiffness, and receptor context can alter invasion or immune behavior even when the original producer remains unresolved (Chen et al., 2022; Sun et al., 2021; Koorman et al., 2022; Kuczek et al., 2019). Most cancer ECM studies establish transcriptional attribution, protein-level compartment assignment, or producer-specific necessity. Far fewer match abundance and deposition context, restore material from the original producer, or exchange it across producers. The remaining task is to identify the proteoform, architecture, or receptor mechanism that explains any persistent difference. Provenance-associated and provenance-independent outcomes are therefore as informative as producer-mediated determinants.

Several unresolved questions define the next phase of ECM provenance research. How often do distinct producers generate different deposited proteoforms from the same ECM gene? When producer identity, spatial location, and co-secretome covary, which has an independent causal effect? How long does production history persist after the producer changes or disappears? Can source-resolved, proteoform-aware ECM proteomics be implemented in patient tissue without a species boundary? Does therapy commonly induce source replacement or relay, and can probes or drugs recognize source-linked proteoforms or assembly states rather than total protein?

Resolving these questions will require complementary methods. Spatial transcriptomics can define candidate states, cell-selective or isotope-based labeling can assign synthesis, imaging can locate mature deposits, and engineered matrices or conditional genetics can test causality. The experimental unit should be a specified product from a specified producer state and production context, not a gene symbol attached to a broad lineage.

The practical value of this framework lies in falsifiability. A well-designed experiment should allow producer identity to lose explanatory power when products are interchangeable and should identify the measurable mediator when a producer-linked difference persists. Applied in this way, testing cellular provenance can sharpen explanations of metastasis, biomarker ambiguity, and therapeutic compensation.

## References

[B1] Acerbi I. Cassereau L. Dean I. Shi Q. Au A. Park C. (2015). Human breast cancer invasion and aggression correlates with ECM stiffening and immune cell infiltration. Integr. Biol. 7, 1120–1134. 10.1039/c5ib00040h 25959051 PMC4593730

[B2] Ahmed D. W. Tan M. L. Liu Y. Gabbard J. Gao E. Roy A. (2025). Local photocrosslinking of native tissue matrix regulates lung epithelial cell mechanosensing and function. Nat. Mater. 24, 1812–1825. 10.1038/s41563-025-02329-0 PMC13020898 40913159

[B3] Alonso-Nocelo M. Ruiz-Cañas L. Sancho P. Görgülü K. Alcalá S. Pedrero C. (2023). Macrophages direct cancer cells through a LOXL2-mediated metastatic cascade in pancreatic ductal adenocarcinoma. Gut 72, 345–359. 10.1136/gutjnl-2021-325564 PMC9872246 35428659

[B4] Alvarez-Castelao B. Schanzenbächer C. T. Hanus C. Glock C. tom Dieck S. Dörrbaum A. R. (2017). Cell-type-specific metabolic labeling of nascent proteomes in vivo. Nat. Biotechnol. 35, 1196–1201. 10.1038/nbt.4016 29106408

[B5] Astrof S. Crowley D. George E. L. Fukuda T. Sekiguchi K. Hanahan D. (2004). Direct test of potential roles of EIIIA and EIIIB alternatively spliced segments of fibronectin in physiological and tumor angiogenesis. Mol. Cell. Biol. 24, 8662–8670. 10.1128/mcb.24.19.8662-8670.2004 15367684 PMC516752

[B6] Attieh Y. Clark A. G. Grass C. Richon S. Pocard M. Mariani P. (2017). Cancer-associated fibroblasts lead tumor invasion through integrin-β3-dependent fibronectin assembly. J. Cell Biol. 216, 3509–3520. 10.1083/jcb.201702033 28931556 PMC5674886

[B7] Bakker G.-J. Weischer S. Ferrer Ortas J. Heidelin J. Andresen V. Beutler M. (2022). Intravital deep-tumor single-beam 3-photon, 4-photon, and harmonic microscopy. eLife 11, e63776. 10.7554/elife.63776 PMC8849342 35166669

[B8] Bansaccal N. Vieugue P. Sarate R. Song Y. Minguijon E. Miroshnikova Y. A. (2023). The extracellular matrix dictates regional competence for tumour initiation. Nature 623, 828–835. 10.1038/s41586-023-06740-y PMC7615367 37968399

[B9] Barney L. E. Hall C. L. Schwartz A. D. Parks A. N. Sparages C. Galarza S. (2020). Tumor cell-organized fibronectin maintenance of a dormant breast cancer population. Sci. Adv. 6, eaaz4157. 10.1126/sciadv.aaz4157 32195352 PMC7065904

[B10] Barrett A. S. Wither M. J. Hill R. C. Dzieciatkowska M. D'Alessandro A. Reisz J. A. (2017). Hydroxylamine chemical digestion for insoluble extracellular matrix characterization. J. Proteome Res. 16, 4177–4184. 10.1021/acs.jproteome.7b00527 28971683 PMC5802359

[B11] Bayer S. V. Grither W. R. Brenot A. Hwang P. Y. Barcus C. E. Ernst M. (2019). DDR2 controls breast tumor stiffness and metastasis by regulating integrin mediated mechanotransduction in CAFs. eLife 8, e45508. 10.7554/elife.45508 31144616 PMC6555593

[B12] Bhattacharjee S. Hamberger F. Ravichandra A. Miller M. Nair A. Affo S. (2021). Tumor restriction by type I collagen opposes tumor-promoting effects of cancer-associated fibroblasts. J. Clin. Investig. 131, e146987. 10.1172/jci146987 PMC8159701 33905375

[B13] Boyce M. Carrico I. S. Ganguli A. S. Yu S.-H. Hangauer M. J. Hubbard S. C. (2011). Metabolic cross-talk allows labeling of O-linked β-N-acetylglucosamine-modified proteins via the N-acetylgalactosamine salvage pathway. Proc. Natl. Acad. Sci. U. S. A. 108, 3141–3146. 10.1073/pnas.1010045108 21300897 PMC3044403

[B14] Boyle S. T. Mittal P. Kaur G. Hoffmann P. Samuel M. S. Klingler-Hoffmann M. (2020). Uncovering tumor-stroma inter-relationships using MALDI mass spectrometry imaging. J. Proteome Res. 19, 4093–4103. 10.1021/acs.jproteome.0c00511 32870688

[B15] Cencioni C. Malatesta S. Vigiano Benedetti V. Licursi V. Perfetto L. Conte F. (2025). The GLP-1R agonist semaglutide reshapes pancreatic cancer associated fibroblasts reducing collagen proline hydroxylation and favoring T lymphocyte infiltration. J. Exp. Clin. Cancer Res. 44, 18. 10.1186/s13046-024-03263-w PMC11744909 39828692

[B16] Chen Y. Kim J. Yang S. Wang H. Wu C.-J. Sugimoto H. (2021). Type I collagen deletion in αSMA+ myofibroblasts augments immune suppression and accelerates progression of pancreatic cancer. Cancer Cell. 39, 548–565.e6. 10.1016/j.ccell.2021.02.007 PMC8423173 33667385

[B17] Chen Y. Yang S. Tavormina J. Tampe D. Zeisberg M. Wang H. (2022). Oncogenic collagen I homotrimers from cancer cells bind to α3β1 integrin and impact tumor microbiome and immunity to promote pancreatic cancer. Cancer Cell. 40, 818–834.e9. 10.1016/j.ccell.2022.06.011 PMC9831277 35868307

[B18] Conklin M. W. Eickhoff J. C. Riching K. M. Pehlke C. A. Eliceiri K. W. Provenzano P. P. (2011). Aligned collagen is a prognostic signature for survival in human breast carcinoma. Am. J. Pathol. 178, 1221–1232. 10.1016/j.ajpath.2010.11.076 21356373 PMC3070581

[B19] Conner S. J. Borges H. B. Guarin J. R. Gerton T. J. Yui A. Salhany K. J. Jr. (2024). Obesity induces temporally regulated alterations in the extracellular matrix that drive breast tumor invasion and metastasis. Cancer Res. 84, 2761–2775. 10.1158/0008-5472.can-23-2526 PMC11908800 38900938

[B20] Costa-Silva B. Aiello N. M. Ocean A. J. Singh S. Zhang H. Thakur B. K. (2015). Pancreatic cancer exosomes initiate pre-metastatic niche formation in the liver. Nat. Cell Biol. 17, 816–826. 10.1038/ncb3169 25985394 PMC5769922

[B21] Crapo P. M. Gilbert T. W. Badylak S. F. (2011). An overview of tissue and whole organ decellularization processes. Biomaterials 32, 3233–3243. 10.1016/j.biomaterials.2011.01.057 21296410 PMC3084613

[B22] Deligne C. Murdamoothoo D. Gammage A. N. Gschwandtner M. Erne W. Loustau T. (2020). Matrix-targeting immunotherapy controls tumor growth and spread by switching macrophage phenotype. Cancer Immunol. Res. 8, 368–382. 10.1158/2326-6066.cir-19-0276 31941671 PMC7611136

[B23] Deryugina E. I. Zajac E. Zilberberg L. Muramatsu T. Joshi G. Dabovic B. (2018). LTBP3 promotes early metastatic events during cancer cell dissemination. Oncogene 37, 1815–1829. 10.1038/s41388-017-0075-1 29348457 PMC5889352

[B24] Di Chiaro P. Nacci L. Arco F. Brandini S. Polletti S. Palamidessi A. (2024). Mapping functional to morphological variation reveals the basis of regional extracellular matrix subversion and nerve invasion in pancreatic cancer. Cancer Cell. 42, 662–681.e10. 10.1016/j.ccell.2024.02.017 38518775

[B25] Di Martino J. S. Nobre A. R. Mondal C. Taha I. Farias E. F. Fertig E. J. (2022). A tumor-derived type III collagen-rich ECM niche regulates tumor cell dormancy. Nat. Cancer 3, 90–107. 10.1038/s43018-021-00291-9 PMC8818089 35121989

[B26] Dong Y. Chen Y. Wang Y. Zhao X. Zi R. Hao J. (2024). Cancer-associated fibroblasts derived fibronectin Extra domain A promotes sorafenib resistance in hepatocellular carcinoma cells by activating SHMT1. Genes Dis. 11, 101330. 10.1016/j.gendis.2024.101330 PMC11402957 39286657

[B27] Eckert M. A. Coscia F. Chryplewicz A. Chang J. W. Hernandez K. M. Pan S. (2019). Proteomics reveals NNMT as a master metabolic regulator of cancer-associated fibroblasts. Nature 569, 723–728. 10.1038/s41586-019-1173-8 31043742 PMC6690743

[B28] Erdogan B. Ao M. White L. M. Means A. L. Brewer B. M. Yang L. (2017). Cancer-associated fibroblasts promote directional cancer cell migration by aligning fibronectin. J. Cell Biol. 216, 3799–3816. 10.1083/jcb.201704053 29021221 PMC5674895

[B29] Erler J. T. Bennewith K. L. Cox T. R. Lang G. Bird D. Koong A. (2009). Hypoxia-induced lysyl oxidase is a critical mediator of bone marrow cell recruitment to form the premetastatic niche. Cancer Cell. 15, 35–44. 10.1016/j.ccr.2008.11.012 19111879 PMC3050620

[B30] Fan W. Adebowale K. Váncza L. Li Y. Rabbi M. F. Kunimoto K. (2024). Matrix viscoelasticity promotes liver cancer progression in the pre-cirrhotic liver. Nature 626, 635–642. 10.1038/s41586-023-06991-9 PMC10866704 38297127

[B31] Feng Y. Ma W. Zang Y. Guo Y. Li Y. Zhang Y. (2024). Spatially organized tumor-stroma boundary determines the efficacy of immunotherapy in colorectal cancer patients. Nat. Commun. 15, 10259. 10.1038/s41467-024-54710-3 PMC11599708 39592630

[B32] Franco-Valls H. Tusquets-Uxó E. Sala L. Val M. Peña R. Iaconcig A. (2023). Formation of an invasion-permissive matrix requires TGFβ/SNAIL1-regulated alternative splicing of fibronectin. Breast Cancer Res. 25, 143. 10.1186/s13058-023-01736-y PMC10647173 37964360

[B33] Fukuda K. Sugihara E. Ohta S. Izuhara K. Funakoshi T. Amagai M. (2015). Periostin is a key niche component for wound metastasis of melanoma. PLoS ONE 10, e0129704. 10.1371/journal.pone.0129704 26083413 PMC4471156

[B34] Ghajar C. M. Peinado H. Mori H. Matei I. R. Evason K. J. Brazier H. (2013). The perivascular niche regulates breast tumour dormancy. Nat. Cell Biol. 15, 807–817. 10.1038/ncb2767 23728425 PMC3826912

[B35] Ghura H. Keimer M. von Au A. Hackl N. Klemis V. Nakchbandi I. A. (2021). Inhibition of fibronectin accumulation suppresses tumor growth. Neoplasia 23, 837–850. 10.1016/j.neo.2021.06.012 PMC8322122 34298233

[B36] Gillot L. Lebeau A. Baudin L. Pottier C. Louis T. Durré T. (2022). Periostin in lymph node pre-metastatic niches governs lymphatic endothelial cell functions and metastatic colonization. Cell. Mol. Life Sci. 79, 295. 10.1007/s00018-022-04262-w PMC9107454 35567669

[B37] Gopal S. Veracini L. Grall D. Butori C. Schaub S. Audebert S. (2017). Fibronectin-guided migration of carcinoma collectives. Nat. Commun. 8, 14105. 10.1038/ncomms14105 28102238 PMC5253696

[B38] Goveia J. Rohlenova K. Taverna F. Treps L. Conradi L.-C. Pircher A. (2020). An integrated gene expression landscape profiling approach to identify lung tumor endothelial cell heterogeneity and angiogenic candidates. Cancer Cell. 37, 21–36.e13. 10.1016/j.ccell.2019.12.001 31935371

[B39] Grünwald B. T. Devisme A. Andrieux G. Vyas F. Aliar K. McCloskey C. W. (2021). Spatially confined sub-tumor microenvironments in pancreatic cancer. Cell. 184, 5577–5592.e18. 10.1016/j.cell.2021.09.022 34644529

[B40] He X.-Y. Gao Y. Ng D. Michalopoulou E. George S. Adrover J. M. (2024). Chronic stress increases metastasis via neutrophil-mediated changes to the microenvironment. Cancer Cell. 42, 474–486.e12. 10.1016/j.ccell.2024.01.013 PMC11300849 38402610

[B41] He D. Wu Q. Tian P. Liu Y. Jia Z. Li Z. (2025). Chemotherapy awakens dormant cancer cells in lung by inducing neutrophil extracellular traps. Cancer Cell. 43, 1622–1636.e7. 10.1016/j.ccell.2025.06.007 40614736

[B42] Heiser C. N. Simmons A. J. Revetta F. McKinley E. T. Ramirez-Solano M. A. Wang J. (2023). Molecular cartography uncovers evolutionary and microenvironmental dynamics in sporadic colorectal tumors. Cell. 186, 5620–5637.e16. 10.1016/j.cell.2023.11.006 PMC10756562 38065082

[B43] Hergueta-Redondo M. Sánchez-Redondo S. Hurtado B. Santos V. Pérez-Martínez M. Ximénez-Embún P. (2025). The impact of a high fat diet and platelet activation on pre-metastatic niche formation. Nat. Commun. 16, 2897. 10.1038/s41467-025-57938-9 PMC11965330 40175356

[B44] Hongu T. Pein M. Insua-Rodríguez J. Gutjahr E. Mattavelli G. Meier J. (2022). Perivascular tenascin C triggers sequential activation of macrophages and endothelial cells to generate a pro-metastatic vascular niche in the lungs. Nat. Cancer 3, 486–504. 10.1038/s43018-022-00353-6 PMC9046090 35469015

[B45] Hoshino A. Costa-Silva B. Shen T.-L. Rodrigues G. Hashimoto A. Tešić Mark M. (2015). Tumour exosome integrins determine organotropic metastasis. Nature 527, 329–335. 10.1038/nature15756 26524530 PMC4788391

[B46] Hoshino A. Costa-Silva B. Shen T.-L. Rodrigues G. Hashimoto A. Tešić Mark M. (2026). Editorial expression of concern: tumour exosome integrins determine organotropic metastasis. Nature 656, E44. 10.1038/s41586-026-10977-8 42557336

[B47] Huang Y.-L. Liang C.-Y. Ritz D. Coelho R. Septiadi D. Estermann M. (2020). Collagen-rich omentum is a premetastatic niche for integrin α2-mediated peritoneal metastasis. eLife 9, e59442. 10.7554/elife.59442 33026975 PMC7541088

[B48] Insua-Rodríguez J. Pein M. Hongu T. Meier J. Descot A. Lowy C. M. (2018). Stress signaling in breast cancer cells induces matrix components that promote chemoresistant metastasis. EMBO Mol. Med. 10, e9003. 10.15252/emmm.201809003 30190333 PMC6180299

[B49] Ippolito L. Duatti A. Iozzo M. Comito G. Pardella E. Lorito N. (2024). Lactate supports cell-autonomous ECM production to sustain metastatic behavior in prostate cancer. EMBO Rep. 25, 3506–3531. 10.1038/s44319-024-00180-z PMC11315984 38907027

[B50] Jachetti E. Caputo S. Mazzoleni S. Brambillasca C. S. Parigi S. M. Grioni M. (2015). Tenascin-C protects cancer stem-like cells from immune surveillance by arresting T-cell activation. Cancer Res. 75, 2095–2108. 10.1158/0008-5472.can-14-2346 25808872

[B51] Jailkhani N. Clauser K. R. Mak H. H. Rickelt S. Tian C. Whittaker C. A. (2023). Proteomic profiling of extracellular matrix components from patient metastases identifies consistently elevated proteins for developing nanobodies that target primary tumors and metastases. Cancer Res. 83, 2052–2065. 10.1158/0008-5472.can-22-1532 PMC10267678 37098922

[B52] Jain S. Rick J. W. Joshi R. S. Beniwal A. Spatz J. Gill S. (2023). Single-cell RNA sequencing and spatial transcriptomics reveal cancer-associated fibroblasts in glioblastoma with protumoral effects. J. Clin. Investig. 133, e147087. 10.1172/jci147087 PMC9974099 36856115

[B53] Jiang S. Li H. Zhang L. Mu W. Zhang Y. Chen T. (2025). Generic diagramming platform (GDP): a comprehensive database of high-quality biomedical graphics. Nucleic Acids Res. 53, D1670–D1676. 10.1093/nar/gkae973 PMC11701665 39470721

[B54] Kaplan R. N. Riba R. D. Zacharoulis S. Bramley A. H. Vincent L. Costa C. (2005). VEGFR1-positive haematopoietic bone marrow progenitors initiate the pre-metastatic niche. Nature 438, 820–827. 10.1038/nature04186 16341007 PMC2945882

[B55] Khan K. A. Caunt Mitzner M. Cruz-Munoz W. Li G. Himmels P. Xu P. (2025). Modulation of fibronectin extracellular matrix enhances anti-tumor efficacy of immune checkpoint blockade. Cell Rep. Med. 6, 102322. 10.1016/j.xcrm.2025.102322 PMC12490255 40925374

[B56] Kieffer Y. Hocine H. R. Gentric G. Pelon F. Bernard C. Bourachot B. (2020). Single-cell analysis reveals fibroblast clusters linked to immunotherapy resistance in cancer. Cancer Discov. 10, 1330–1351. 10.1158/2159-8290.cd-19-1384 32434947

[B57] Kim K.-E. Park I. Kim J. Kang M.-G. Choi W. G. Shin H. (2021). Dynamic tracking and identification of tissue-specific secretory proteins in the circulation of live mice. Nat. Commun. 12, 5204. 10.1038/s41467-021-25546-y PMC8410947 34471136

[B58] Koorman T. Jansen K. A. Khalil A. Haughton P. D. Visser D. Rätze M. A. K. (2022). Spatial collagen stiffening promotes collective breast cancer cell invasion by reinforcing extracellular matrix alignment. Oncogene 41, 2458–2469. 10.1038/s41388-022-02258-1 PMC9033577 35292774

[B59] Krasny L. Paul A. Wai P. Howard B. A. Natrajan R. C. Huang P. H. (2016). Comparative proteomic assessment of matrisome enrichment methodologies. Biochem. J. 473, 3979–3995. 10.1042/bcj20160686 27589945 PMC5095915

[B60] Krasny L. Bland P. Burns J. Lima N. C. Harrison P. T. Pacini L. (2020). A mouse SWATH-MS reference spectral library enables deconvolution of species-specific proteomic alterations in human tumour xenografts. Dis. Model. Mech. 13, dmm044586. 10.1242/dmm.044586 32493768 PMC7375474

[B61] Kuczek D. E. Larsen A. M. H. Thorseth M.-L. Carretta M. Kalvisa A. Siersbæk M. S. (2019). Collagen density regulates the activity of tumor-infiltrating T cells. J. Immunother. Cancer 7, 68. 10.1186/s40425-019-0556-6 30867051 PMC6417085

[B62] Lambert A. W. Wong C. K. Ozturk S. Papageorgis P. Raghunathan R. Alekseyev Y. (2016). Tumor cell-derived periostin regulates cytokines that maintain breast cancer stem cells. Mol. Cancer Res. 14, 103–113. 10.1158/1541-7786.mcr-15-0079 26507575 PMC4715959

[B63] Lee Y.-C. Lin S.-C. Yu G. Zhu M. Song J. H. Rivera K. (2022). Prostate tumor-induced stromal reprogramming generates tenascin C that promotes prostate cancer metastasis through YAP/TAZ inhibition. Oncogene 41, 757–769. 10.1038/s41388-021-02131-7 PMC8818031 34845375

[B64] Lee H. J. Park S. W. Lee J. H. Chang S. Y. Oh S. M. Mun S. (2025). Differential cellular origins of the extracellular matrix of tumor and normal tissues according to colorectal cancer subtypes. Br. J. Cancer 132, 770–782. 10.1038/s41416-025-02964-z PMC12041468 40032993

[B65] Leng Y. Lipp S. N. Bu Y. Larson H. Jacobson K. R. Colling H. A. (2025). Extracellular matrix deposition precedes muscle-tendon integration during murine forelimb morphogenesis. Commun. Biol. 8, 1202. 10.1038/s42003-025-08653-0 PMC12343929 40796656

[B66] Levental K. R. Yu H. Kass L. Lakins J. N. Egeblad M. Erler J. T. (2009). Matrix crosslinking forces tumor progression by enhancing integrin signaling. Cell. 139, 891–906. 10.1016/j.cell.2009.10.027 19931152 PMC2788004

[B67] Leverton L. Pally D. Jones A. Therol C. Ricard-Blum S. Naba A. (2026). SNED1 fibrillar assembly in the extracellular matrix requires fibronectin and collagen I. J. Cell Sci. 139, jcs264884. 10.1242/jcs.264884 PMC13403931 42473797

[B68] Lewinska M. Zhuravleva E. Satriano L. Martinez M. B. Bhatt D. K. Oliveira D. V. N. P. (2024). Fibroblast-derived lysyl oxidase increases oxidative phosphorylation and stemness in cholangiocarcinoma. Gastroenterology 166, 886–901.e7. 10.1053/j.gastro.2023.11.302 38096955

[B69] Li W. Dasgupta A. Yang K. Wang S. Hemandhar-Kumar N. Chepyala S. R. (2025). Turnover atlas of proteome and phosphoproteome across mouse tissues and brain regions. Cell. 188, 2267–2287.e21. 10.1016/j.cell.2025.02.021 PMC12033170 40118046

[B70] Li A. Wang Y. Bai H. Xie X. Xu B. Wang Y. (2026). Macrophage-derived fibronectin suppresses antitumor immunity via tissue stiffening and immunosuppressive cell induction in cancer mouse models. Nat. Commun. 17, 7296. 10.1038/s41467-026-73287-7 PMC13402297 42173863

[B71] Ling Z. Niego B. Li Q. Bhattaram D. Serna Villa V. Hu M. Y. (2025). Chemoselective characterization of new extracellular matrix deposition in bioengineered tumor tissues. Adv. Mater. 37, e05445. 10.1002/adma.202505445 PMC12500236 40913457

[B72] Ling X. Zhang Y. Zhu L. Xu Z. Bao Y. Lin H. (2026). Tumor cell-derived HSP47 promotes pancreatic cancer metastasis via homotrimeric collagen-mediated M2 macrophage polarization. Cell. Signal. 145, 112589. 10.1016/j.cellsig.2026.112589 42128153

[B73] Liu Y. Beyer A. Aebersold R. (2016). On the dependency of cellular protein levels on mRNA abundance. Cell. 165, 535–550. 10.1016/j.cell.2016.03.014 27104977

[B74] Liu X. Li J. Yang X. Li X. Kong J. Qi D. (2023). Carcinoma-associated fibroblast-derived lysyl oxidase-rich extracellular vesicles mediate collagen crosslinking and promote epithelial-mesenchymal transition via p-FAK/p-paxillin/YAP signaling. Int. J. Oral Sci. 15, 32. 10.1038/s41368-023-00236-1 PMC10397209 37532712

[B75] Loustau T. Mitrentsi I. Wang N. Spenlé C. Pavlidaki A. Tranchant T. (2026). Tenascin-C orchestrates radiotherapy-induced head and neck tumor regression. EMBO Mol. Med. 18, 1707–1743. 10.1038/s44321-026-00406-8 PMC13179387 41917325

[B76] Lubosch A. Pitt L. Zoeller C. Wirth F. Exner T. Steigenberger B. (2025). The immune response against cancer is modulated by stromal cell fibronectin. Neoplasia 67, 101196. 10.1016/j.neo.2025.101196 PMC12266377 40592236

[B77] Lugano R. Vemuri K. Yu D. Bergqvist M. Smits A. Essand M. (2018). CD93 promotes β1 integrin activation and fibronectin fibrillogenesis during tumor angiogenesis. J. Clin. Investig. 128, 3280–3297. 10.1172/jci97459 29763414 PMC6063507

[B78] Lyons T. R. O'Brien J. Borges V. F. Conklin M. W. Keely P. J. Eliceiri K. W. (2011). Postpartum mammary gland involution drives progression of ductal carcinoma in situ through collagen and COX-2. Nat. Med. 17, 1109–1115. 10.1038/nm.2416 PMC3888478 21822285

[B79] Macdonald J. K. Taylor H. B. Wang M. Delacourt A. Edge C. Lewin D. N. (2024). The spatial extracellular proteomic tumor microenvironment distinguishes molecular subtypes of hepatocellular carcinoma. J. Proteome Res. 23, 3791–3805. 10.1021/acs.jproteome.4c00099 PMC11385377 38980715

[B80] Malanchi I. Santamaria-Martínez A. Susanto E. Peng H. Lehr H.-A. Delaloye J.-F. (2012). Interactions between cancer stem cells and their niche govern metastatic colonization. Nature 481, 85–89. 10.1038/nature10694 22158103

[B81] Malik G. Knowles L. M. Dhir R. Xu S. Yang S. Ruoslahti E. (2010). Plasma fibronectin promotes lung metastasis by contributions to fibrin clots and tumor cell invasion. Cancer Res. 70, 4327–4334. 10.1158/0008-5472.can-09-3312 20501851 PMC2880218

[B82] Mariathasan S. Turley S. J. Nickles D. Castiglioni A. Yuen K. Wang Y. (2018). TGFβ attenuates tumour response to PD-L1 blockade by contributing to exclusion of T cells. Nature 554, 544–548. 10.1038/nature25501 29443960 PMC6028240

[B83] McCabe M. C. Schmitt L. R. Hill R. C. Dzieciatkowska M. Maslanka M. Daamen W. F. (2021). Evaluation and refinement of sample preparation methods for extracellular matrix proteome coverage. Mol. Cell. Proteomics 20, 100079. 10.1016/j.mcpro.2021.100079 PMC8188056 33845168

[B84] Mirzaei R. Sarkar S. Dzikowski L. Rawji K. S. Khan L. Faissner A. (2018). Brain tumor-initiating cells export tenascin-C associated with exosomes to suppress T cell activity. Oncoimmunology 7, e1478647. 10.1080/2162402x.2018.1478647 30288344 PMC6169571

[B85] Murgai M. Ju W. Eason M. Kline J. Beury D. W. Kaczanowska S. (2017). KLF4-dependent perivascular cell plasticity mediates pre-metastatic niche formation and metastasis. Nat. Med. 23, 1176–1190. 10.1038/nm.4400 28920957 PMC5724390

[B86] Murphy P. A. Begum S. Hynes R. O. (2015). Tumor angiogenesis in the absence of fibronectin or its cognate integrin receptors. PLoS ONE 10, e0120872. 10.1371/journal.pone.0120872 25807551 PMC4373772

[B87] Naba A. (2023). Ten years of extracellular matrix proteomics: accomplishments, challenges, and future perspectives. Mol. Cell. Proteomics 22, 100528. 10.1016/j.mcpro.2023.100528 PMC10152135 36918099

[B88] Naba A. Clauser K. R. Hoersch S. Liu H. Carr S. A. Hynes R. O. (2012). The matrisome: in silico definition and in vivo characterization by proteomics of normal and tumor extracellular matrices. Mol. Cell. Proteomics 11, 014647. 10.1074/mcp.m111.014647 22159717 PMC3322572

[B89] Naba A. Clauser K. R. Lamar J. M. Carr S. A. Hynes R. O. (2014). Extracellular matrix signatures of human mammary carcinoma identify novel metastasis promoters. eLife 3, e01308. 10.7554/elife.01308 24618895 PMC3944437

[B90] Nicolas-Boluda A. Vaquero J. Vimeux L. Guilbert T. Barrin S. Kantari-Mimoun C. (2021). Tumor stiffening reversion through collagen crosslinking inhibition improves T cell migration and anti-PD-1 treatment. eLife 10, e58688. 10.7554/elife.58688 PMC8203293 34106045

[B91] Null J. L. Kim D. J. McCann J. V. Pramoonjago P. Fox J. W. Zeng J. (2023). Periostin+ stromal cells guide lymphovascular invasion by cancer cells. Cancer Res. 83, 2105–2122. 10.1158/0008-5472.can-22-2412 PMC10330490 37205636

[B92] O'Connell J. T. Sugimoto H. Cooke V. G. MacDonald B. A. Mehta A. I. LeBleu V. S. (2011). VEGF-A and tenascin-C produced by S100A4+ stromal cells are important for metastatic colonization. Proc. Natl. Acad. Sci. U. S. A. 108, 16002–16007. 10.1073/pnas.1109493108 PMC3179047 21911392

[B93] O'Reilly M. S. Boehm T. Shing Y. Fukai N. Vasios G. Lane W. S. (1997). Endostatin: an endogenous inhibitor of angiogenesis and tumor growth. Cell. 88, 277–285. 10.1016/s0092-8674(00)81848-6 9008168

[B94] Öhlund D. Handly-Santana A. Biffi G. Elyada E. Almeida A. S. Ponz-Sarvise M. (2017). Distinct populations of inflammatory fibroblasts and myofibroblasts in pancreatic cancer. J. Exp. Med. 214, 579–596. 10.1084/jem.20162024 28232471 PMC5339682

[B95] Ohta Y. Fujii M. Takahashi S. Takano A. Nanki K. Matano M. (2022). Cell-matrix interface regulates dormancy in human colon cancer stem cells. Nature 608, 784–794. 10.1038/s41586-022-05043-y 35798028

[B96] Oskarsson T. Acharyya S. Zhang X.H.-F. Vanharanta S. Tavazoie S. F. Morris P. G. (2011). Breast cancer cells produce tenascin C as a metastatic niche component to colonize the lungs. Nat. Med. 17, 867–874. 10.1038/nm.2379 21706029 PMC4020577

[B97] Pally D. Kapoor N. Naba A. (2025). The novel ECM protein SNED1 mediates cell adhesion via the RGD-binding integrins α5β1 and αvβ3. J. Cell Sci. 138, JCS263479. 10.1242/jcs.263479 PMC11828466 39713860

[B98] Pally D. Leverton L. Jones A. Naba A. (2026). SNED1 modulates ECM architecture and cell proliferation via its LDV integrin-binding motif. J. Cell Sci. 139, jcs264885. 10.1242/jcs.264885 PMC13403930 42439511

[B99] Palumbo J. S. Kombrinck K. W. Drew A. F. Grimes T. S. Kiser J. H. Degen J. L. (2000). Fibrinogen is an important determinant of the metastatic potential of circulating tumor cells. Blood 96, 3302–3309. 10.1182/blood.V96.10.3302 11071621

[B100] Palumbo J. S. Potter J. M. Kaplan L. S. Talmage K. Jackson D. G. Degen J. L. (2002). Spontaneous hematogenous and lymphatic metastasis, but not primary tumor growth or angiogenesis, is diminished in fibrinogen-deficient mice. Cancer Res. 62, 6966–6972. 12460914

[B101] Palumbo J. S. Talmage K. E. Massari J. V. La Jeunesse C. M. Flick M. J. Kombrinck K. W. (2005). Platelets and fibrin(ogen) increase metastatic potential by impeding natural killer cell-mediated elimination of tumor cells. Blood 105, 178–185. 10.1182/blood-2004-06-2272 15367435

[B102] Papanicolaou M. Parker A. L. Yam M. Filipe E. C. Wu S. Z. Chitty J. L. (2022). Temporal profiling of the breast tumour microenvironment reveals collagen XII as a driver of metastasis. Nat. Commun. 13, 4587. 10.1038/s41467-022-32255-7 PMC9357007 35933466

[B103] Parikainen M. Suwal U. Rappu P. Heino J. Sahlgren C. M. (2026). Jagged1 regulates extracellular matrix deposition and remodeling in triple-negative breast cancer. Sci. Adv. 12, eaea9562. 10.1126/sciadv.aea9562 PMC12998526 41849611

[B104] Pascual G. Domínguez D. Elosúa-Bayes M. Beckedorff F. Laudanna C. Bigas C. (2021). Dietary palmitic acid promotes a prometastatic memory via schwann cells. Nature 599, 485–490. 10.1038/s41586-021-04075-0 34759321

[B105] Peinado H. Alečković M. Lavotshkin S. Matei I. Costa-Silva B. Moreno-Bueno G. (2012). Melanoma exosomes educate bone marrow progenitor cells toward a pro-metastatic phenotype through MET. Nat. Med. 18, 883–891. 10.1038/nm.2753 22635005 PMC3645291

[B106] Peng D. H. Rodriguez B. L. Diao L. Chen L. Wang J. Byers L. A. (2020). Collagen promotes anti-PD-1/PD-L1 resistance in cancer through LAIR1-dependent CD8+ T cell exhaustion. Nat. Commun. 11, 4520. 10.1038/s41467-020-18298-8 32908154 PMC7481212

[B107] Pereira B. A. Ritchie S. Chambers C. R. Gordon K. A. Magenau A. Murphy K. J. (2024). Temporally resolved proteomics identifies nidogen-2 as a cotarget in pancreatic cancer that modulates fibrosis and therapy response. Sci. Adv. 10, eadl1197. 10.1126/sciadv.adl1197 PMC11221519 38959305

[B108] Pospelov A. D. Kutova O. M. Efremov Y. M. Nekrasova A. A. Trushina D. B. Gefter S. D. (2023). Breast cancer cell type and biomechanical properties of decellularized mouse organs drives tumor cell colonization. Cells 12, 2030. 10.3390/cells12162030 PMC10453279 37626840

[B109] Provenzano P. P. Eliceiri K. W. Campbell J. M. Inman D. R. White J. G. Keely P. J. (2006). Collagen reorganization at the tumor-stromal interface facilitates local invasion. BMC Med. 4, 38. 10.1186/1741-7015-4-38 17190588 PMC1781458

[B110] Provenzano P. P. Inman D. R. Eliceiri K. W. Knittel J. G. Yan L. Rueden C. T. (2008). Collagen density promotes mammary tumor initiation and progression. BMC Med. 6, 11. 10.1186/1741-7015-6-11 18442412 PMC2386807

[B111] Puttock E. H. Tyler E. J. Manni M. Maniati E. Butterworth C. Burger Ramos M. (2023). Extracellular matrix educates an immunoregulatory tumor macrophage phenotype found in ovarian cancer metastasis. Nat. Commun. 14, 2514. 10.1038/s41467-023-38093-5 PMC10185550 37188691

[B112] Risom T. Glass D. R. Averbukh I. Liu C. C. Baranski A. Kagel A. (2022). Transition to invasive breast cancer is associated with progressive changes in the structure and composition of tumor stroma. Cell. 185, 299–310.e18. 10.1016/j.cell.2021.12.023 PMC8792442 35063072

[B113] Rosin N. L. Winstone T. M. L. Kelley M. Biernaskie J. Dufour A. Orton D. J. (2024). Targeted proteomic approach for quantification of collagen type I and type III in formalin-fixed paraffin-embedded tissue. Sci. Rep. 14, 17769. 10.1038/s41598-024-68377-9 PMC11294326 39090134

[B114] Rossnagl S. Altrock E. Sens C. Kraft S. Rau K. Milsom M. D. (2016). EDA-Fibronectin originating from osteoblasts inhibits the immune response against cancer. PLoS Biol. 14, e1002562. 10.1371/journal.pbio.1002562 27653627 PMC5031442

[B115] Rujchanarong D. Lefler J. Saunders J. E. Pippin S. Spruill L. Bethard J. R. (2021). Defining the tumor microenvironment by integration of immunohistochemistry and extracellular matrix targeted imaging mass spectrometry. Cancers 13, 4419. 10.3390/cancers13174419 PMC8430776 34503228

[B116] Rybak J.-N. Roesli C. Kaspar M. Villa A. Neri D. (2007). The extra-domain A of fibronectin is a vascular marker of solid tumors and metastases. Cancer Res. 67, 10948–10957. 10.1158/0008-5472.can-07-1436 18006840

[B117] Saatci O. Kaymak A. Raza U. Ersan P. G. Akbulut O. Banister C. E. (2020). Targeting lysyl oxidase (LOX) overcomes chemotherapy resistance in triple negative breast cancer. Nat. Commun. 11, 2416. 10.1038/s41467-020-16199-4 32415208 PMC7229173

[B118] Shi Z. Mao B. Chen X. Hao P. Guo S. (2023). Mouse stromal cells confound proteomic characterization and quantification of xenograft models. Cancer Res. Commun. 3, 202–214. 10.1158/2767-9764.crc-22-0431 PMC10035517 36968139

[B119] Shinde A. Libring S. Alpsoy A. Abdullah A. Schaber J. A. Solorio L. (2018). Autocrine fibronectin inhibits breast cancer metastasis. Mol. Cancer Res. 16, 1579–1589. 10.1158/1541-7786.mcr-18-0151 29934326 PMC6511995

[B120] Song L. Xing S. Li P. Cao Y. Ma H. (2026). A clinically relevant SLC2A1-associated malignant epithelial cell state predicts prognosis and immunotherapy response in lung adenocarcinoma. Funct. Integr. Genomics 26, 112. 10.1007/s10142-026-01897-x 42209795 PMC13219086

[B121] Spenlé C. Loustau T. Burckel H. Riegel G. Abou Faycal C. Li C. (2021). Impact of tenascin-C on radiotherapy in a novel syngeneic oral squamous cell carcinoma model with spontaneous dissemination to the lymph nodes. Front. Immunol. 12, 636108. 10.3389/fimmu.2021.636108 34290694 PMC8287883

[B122] Sreekumar A. Lu M. Choudhury B. Pan T.-C. Pant D. K. Lawrence-Paul M. R. (2024). B3GALT6 promotes dormant breast cancer cell survival and recurrence by enabling heparan sulfate-mediated FGF signaling. Cancer Cell. 42, 52–69.e7. 10.1016/j.ccell.2023.11.008 38065100 PMC10872305

[B123] Stewart D. C. Brisson B. K. Dekky B. Berger A. C. Yen W. Mauldin E. A. (2024). Prognostic and therapeutic implications of tumor-restrictive type III collagen in the breast cancer microenvironment. Npj Breast Cancer 10, 86. 10.1038/s41523-024-00690-y PMC11447064 39358397

[B124] Su H. Yang F. Fu R. Trinh B. Sun N. Liu J. (2022). Collagenolysis-dependent DDR1 signalling dictates pancreatic cancer outcome. Nature 610, 366–372. 10.1038/s41586-022-05169-z 36198801 PMC9588640

[B125] Sudhakar A. Sugimoto H. Yang C. Lively J. Zeisberg M. Kalluri R. (2003). Human tumstatin and human endostatin exhibit distinct antiangiogenic activities mediated by αvβ3 and α5β1 integrins. Proc. Natl. Acad. Sci. U. S. A. 100, 4766–4771. 10.1073/pnas.0730882100 12682293 PMC153630

[B126] Sun X. Wu B. Chiang H.-C. Deng H. Zhang X. Xiong W. (2021). Tumour DDR1 promotes collagen fibre alignment to instigate immune exclusion. Nature 599, 673–678. 10.1038/s41586-021-04057-2 34732895 PMC8839149

[B127] Swietlik J. J. Bärthel S. Falcomatà C. Fink D. Sinha A. Cheng J. (2023). Cell-selective proteomics segregates pancreatic cancer subtypes by extracellular proteins in tumors and circulation. Nat. Commun. 14, 2642. 10.1038/s41467-023-38171-8 37156840 PMC10167354

[B128] Tang X. Khan H. Niewola-Staszkowska K. Wuest F. Brindley D. N. (2025). Inhibition of autotaxin activity with IOA-289 decreases fibrosis in mouse E0771 breast tumors. Int. J. Cancer 157, 1205–1217. 10.1002/ijc.35471 40345856 PMC12280849

[B129] Tian C. Huang Y. Clauser K. R. Rickelt S. Lau A. N. Carr S. A. (2021). Suppression of pancreatic ductal adenocarcinoma growth and metastasis by fibrillar collagens produced selectively by tumor cells. Nat. Commun. 12, 2328. 10.1038/s41467-021-22490-9 33879793 PMC8058088

[B130] Tillotson J. Aryal B. Lai L. Beaver J. A. Rao V. A. (2023). Differential protein citrullination in human ER- and ER+ tumor and adjacent healthy breast tissue. Biochemistry 62, 893–898. 10.1021/acs.biochem.2c00551 36757899 PMC9948284

[B131] Tong Y. Qi Y. Xiong G. Li J. Scott T. L. Chen J. (2023). The PLOD2/succinate axis regulates the epithelial-mesenchymal plasticity and cancer cell stemness. Proc. Natl. Acad. Sci. U. S. A. 120, e2214942120. 10.1073/pnas.2214942120 37155842 PMC10194013

[B132] Urtreger A. Porro F. Puricelli L. Werbajh S. Baralle F. E. Bal de Kier Joffé E. (1998). Expression of RGD minus fibronectin that does not form extracellular matrix fibrils is sufficient to decrease tumor metastasis. Int. J. Cancer 78, 233–241. 9754657 10.1002/(sici)1097-0215(19981005)78:2<233::aid-ijc18>3.0.co;2-b

[B133] Vallet S. D. Davis M. N. Barqué A. Thahab A. H. Ricard-Blum S. Naba A. (2021). Computational and experimental characterization of the novel ECM glycoprotein SNED1 and prediction of its interactome. Biochem. J. 478, 1413–1434. 10.1042/bcj20200675 33724335

[B134] Van Passen C. Krug J. Weiß L. Abdou M. M. Tripal P. Schmid B. (2025). Integrin β6 expression in colorectal cancer cells promotes liver metastasis through enhanced adhesion to endothelial fibronectin. Int. J. Cancer 157, 1481–1495. 10.1002/ijc.35504 40488391 PMC12334909

[B135] van Tienderen G. S. Conboy J. Muntz I. Willemse J. Tieleman J. Monfils K. (2023). Tumor decellularization reveals proteomic and mechanical characteristics of the extracellular matrix of primary liver cancer. Biomater. Adv 146, 213289. 10.1016/j.bioadv.2023.213289 36724550

[B136] Verginadis I. I. Avgousti H. Monslow J. Skoufos G. Chinga F. Kim K. (2022). A stromal integrated stress response activates perivascular cancer-associated fibroblasts to drive angiogenesis and tumour progression. Nat. Cell Biol. 24, 940–953. 10.1038/s41556-022-00918-8 35654839 PMC9203279

[B137] Vogel C. Marcotte E. M. (2012). Insights into the regulation of protein abundance from proteomic and transcriptomic analyses. Nat. Rev. Genet. 13, 227–232. 10.1038/nrg3185 22411467 PMC3654667

[B138] von Au A. Vasel M. Kraft S. Sens C. Hackl N. Marx A. (2013). Circulating fibronectin controls tumor growth. Neoplasia 15, 925–938. 10.1593/neo.13762 23908593 PMC3730044

[B139] Wang Z. Xiong S. Mao Y. Chen M. Ma X. Zhou X. (2016). Periostin promotes immunosuppressive premetastatic niche formation to facilitate breast tumour metastasis. J. Pathol. 239, 484–495. 10.1002/path.4747 27193093

[B140] Wang X. Mooradian A. D. Erdmann-Gilmore P. Zhang Q. Viner R. Davies S. R. (2017). Breast tumors educate the proteome of stromal tissue in an individualized but coordinated manner. Sci. Signal. 10, eaam8065. 10.1126/scisignal.aam8065 28790197 PMC5712229

[B141] Wang X. Niu J. Qian S. Shen S. Straubinger R. M. Qu J. (2023). Species-deconvolved proteomics for in situ investigation of tumor-stroma interactions after treatment of pancreatic cancer patient-derived xenografts with combined gemcitabine and paclitaxel. J. Proteome Res. 22, 2436–2449. 10.1021/acs.jproteome.3c00164 37311110 PMC10561664

[B142] Wang L. Li C. Zhan H. Li S. Zeng K. Xu C. (2024). Targeting the HSP47-collagen axis inhibits brain metastasis by reversing M2 microglial polarization and restoring anti-tumor immunity. Cell Rep. Med. 5, 101533. 10.1016/j.xcrm.2024.101533 38744278 PMC11149409

[B143] Wang W. Hu K. Xue J. Chen J. Du X. Zhao T. (2025). In vivo FAP-CAR macrophages enhance chemotherapy and immunotherapy against pancreatic cancer by removing the fibrosis barrier. J. Control. Release 384, 113888. 10.1016/j.jconrel.2025.113888 40425095

[B144] Watson S. S. Duc B. Kang Z. de Tonnac A. Eling N. Font L. (2024). Microenvironmental reorganization in brain tumors following radiotherapy and recurrence revealed by hyperplexed immunofluorescence imaging. Nat. Commun. 15, 3226. 10.1038/s41467-024-47185-9 38622132 PMC11018859

[B145] Wei W. Riley N. M. Yang A. C. Kim J. T. Terrell S. M. Li V. L. (2021). Cell type-selective secretome profiling in vivo. Nat. Chem. Biol. 17, 326–334. 10.1038/s41589-020-00698-y 33199915 PMC7904581

[B146] Wu S. Z. Roden D. L. Wang C. Holliday H. Harvey K. Cazet A. S. (2020). Stromal cell diversity associated with immune evasion in human triple-negative breast cancer. EMBO J. 39, e104063. 10.15252/embj.2019104063 32790115 PMC7527929

[B147] Wu F. Tabang D. N. Wang D. Odorico J. S. Li L. (2025). Proteome-wide investigation of proline hydroxylation in pancreatic ductal adenocarcinoma using DiLeu isobaric labeling strategy. Mol. Cell. Proteomics 24, 100969. 10.1016/j.mcpro.2025.100969 40216311 PMC12275936

[B148] Xiong G. Stewart R. L. Chen J. Gao T. Scott T. L. Samayoa L. M. (2018). Collagen prolyl 4-hydroxylase 1 is essential for HIF-1α stabilization and TNBC chemoresistance. Nat. Commun. 9, 4456. 10.1038/s41467-018-06893-9 30367042 PMC6203834

[B149] Xiong G. Chen J. Zhang G. Wang S. Kawasaki K. Zhu J. (2020). Hsp47 promotes cancer metastasis by enhancing collagen-dependent cancer cell-platelet interaction. Proc. Natl. Acad. Sci. U. S. A. 117, 3748–3758. 10.1073/pnas.1911951117 32015106 PMC7035603

[B150] Yang X. Chen X. Zhang S. Fan W. Zhong C. Liu T. (2023). Collagen 1-mediated CXCL1 secretion in tumor cells activates fibroblasts to promote radioresistance of esophageal cancer. Cell Rep. 42, 113270. 10.1016/j.celrep.2023.113270 37851572

[B151] Yang J. Bahcecioglu G. Ronan G. Zorlutuna P. (2024). Aged breast matrix bound vesicles promote breast cancer invasiveness. Biomaterials 306, 122493. 10.1016/j.biomaterials.2024.122493 38330741 PMC11202350

[B152] Zaccara S. Ries R. J. Jaffrey S. R. (2019). Reading, writing and erasing mRNA methylation. Nat. Rev. Mol. Cell Biol. 20, 608–624. 10.1038/s41580-019-0168-5 31520073

[B153] Zhang Y. Cedervall J. Hamidi A. Herre M. Viitaniemi K. D'Amico G. (2020). Platelet-specific PDGFB ablation impairs tumor vessel integrity and promotes metastasis. Cancer Res. 80, 3345–3358. 10.1158/0008-5472.can-19-3533 32586981

[B154] Zhu T. Alves S. M. Adamo A. Wen X. Corn K. C. Shostak A. (2024). Mammary tissue-derived extracellular matrix hydrogels reveal the role of irradiation in driving a pro-tumor and immunosuppressive microenvironment. Biomaterials 308, 122531. 10.1016/j.biomaterials.2024.122531 38531198 PMC11065579

